# Influenza Vaccine Immune Response in Patients With High-Risk Cardiovascular Disease: A Secondary Analysis of the INVESTED Randomized Clinical Trial

**DOI:** 10.1001/jamacardio.2024.0468

**Published:** 2024-04-07

**Authors:** Alexander Peikert, Brian L. Claggett, Jacob A. Udell, Jacob Joseph, Sheila M. Hegde, KyungMann Kim, Lu Mao, Tuo Wang, Thomas C. Havighurst, Michael E. Farkouh, Deepak L. Bhatt, Matthew C. Tattersall, Lawton S. Cooper, Scott D. Solomon, Orly Vardeny

**Affiliations:** 1 Cardiovascular Division, Brigham and Women’s Hospital, Harvard Medical School, Boston, Massachusetts; 2 Peter Munk Cardiac Centre, University Health Network and Women’s College Hospital, University of Toronto, Toronto, Ontario, Canada; 3 Department of Medicine, VA Providence Healthcare System and Brown University, Providence, Rhode Island; 4 Department of Biostatistics and Medical Informatics, University of Wisconsin–Madison, Madison; 5 Cedars-Sinai Health System, Los Angeles, California; 6 Mount Sinai Heart, Icahn School of Medicine at Mount Sinai Health System, New York, New York; 7 Division of Cardiovascular Medicine, Department of Medicine, University of Wisconsin–Madison, Madison; 8 National Heart, Lung, and Blood Institute, Bethesda, Maryland; 9 Department of Medicine, University of Minnesota, Minneapolis VA Health Care System, Minneapolis; 10 University Heart Center Graz, Department of Cardiology, Medical University of Graz, Graz, Austria

## Abstract

**Importance:**

High-dose trivalent compared with standard-dose quadrivalent influenza vaccine did not significantly reduce all-cause mortality or cardiopulmonary hospitalizations in patients with high-risk cardiovascular disease in the INVESTED trial. Whether humoral immune response to influenza vaccine is associated with clinical outcomes is unknown.

**Objective:**

To examine the antibody response to high-dose trivalent compared with standard-dose quadrivalent inactivated influenza vaccine and its associations with clinical outcomes.

**Design, Setting, and Participants:**

This secondary analysis is a prespecified analysis of the immune response substudy of the randomized, double-blind, active-controlled INVESTED trial, which was conducted at 157 sites in the United States and Canada over 3 influenza seasons between September 2016 and January 2019. Antibody titers were determined by hemagglutination inhibition assays at randomization and 4 weeks during the 2017-2018 and 2018-2019 seasons. Eligibility criteria included recent acute myocardial infarction or heart failure hospitalization and at least 1 additional risk factor. Data were analyzed from February 2023 to June 2023.

**Main Outcomes and Measures:**

Mean antibody titer change, seroprotection (antibody titer level ≥1:40) and seroconversion (≥4-fold increase in titer) at 4 weeks, and the association between seroconversion status and the risk for adverse clinical outcomes.

**Interventions:**

High-dose trivalent or standard-dose quadrivalent inactivated influenza vaccine, with revaccination up to 3 seasons.

**Results:**

Antibody data were available for 658 of 5260 randomized participants (12.5%; mean [SD] age, 66.2 [11.4] years; 507 male [77.1%], 151 female [22.9%]; 348 with heart failure [52.9%]). High-dose vaccine was associated with an increased magnitude in antibody titers for A/H1N1, A/H3N2, and B-type antigens compared with standard dose. More than 92% of all participants achieved seroprotection for each of the contained antigens, while seroconversion rates were higher in participants who received high-dose vaccine. Seroconversion for any antigen was not associated with the risk for cardiopulmonary hospitalizations or all-cause mortality (hazard ratio, 1.09; 95% CI, 0.79-1.53; *P* = .59), irrespective of randomized treatment (*P* = .38 for interaction).

**Conclusions and Relevance:**

High-dose vaccine elicited a more robust humoral response in patients with heart failure or prior myocardial infarction enrolled in the INVESTED trial, with no association between seroconversion status and the risk for cardiopulmonary hospitalizations or all-cause mortality. Vaccination to prevent influenza remains critical in high-risk populations.

**Trial Registration:**

ClinicalTrials.gov Identifier: NCT02787044

## Key Points

**Question:**

Does the antibody response to high-dose trivalent compared with standard-dose quadrivalent influenza vaccine differ among patients with recent myocardial infarction or hospitalization for heart failure, and are immune responses associated with adverse clinical outcomes?

**Findings:**

In this prespecified secondary analysis of the INVESTED trial, high-dose vaccine resulted in a stronger humoral response and higher rates of seroconversion, with no association between seroconversion status and the risk for cardiopulmonary hospitalizations or all-cause mortality, regardless of vaccine dose.

**Meaning:**

High-dose influenza vaccine confers a more robust humoral response in patients with high-risk cardiovascular disease, whereas antibody responses did not influence the risk of adverse clinical outcomes.

## Teaser

This secondary analysis of a randomized clinical trial examines the antibody response to high-dose trivalent compared with standard-dose quadrivalent inactivated influenza vaccine in patients with a recent acute myocardial infarction or heart failure hospitalization.

## Introduction

Patients with cardiovascular disease are at increased risk for influenza-related complications.^1,2^ Influenza vaccination has been shown to reduce adverse cardiovascular events and is recommended by current guidelines in high-risk patients.^3,4,5,6^ While the protection elicited by vaccines is presumed to be attenuated in adults with older age or comorbidities such as cardiovascular disease or heart failure (HF) due to reduced antibody response, vaccine formulations with higher hemagglutinin concentrations may improve efficacy and immunogenicity in these patient groups.^7,8,9,10^ In the INVESTED (Influenza Vaccine to Effectively Stop Cardio Thoracic Events and Decompensated Heart Failure) trial, high-dose trivalent inactivated influenza vaccine did not reduce all-cause mortality or cardiopulmonary hospitalizations compared with quadrivalent standard-dose vaccine in patients with recent HF hospitalization or acute myocardial infarction (MI).^11^ Whether the humoral response may differ by vaccine dose in patients with high-risk cardiovascular disease and is associated with clinical events is unknown. In this prespecified analysis of the INVESTED trial, we examined the antibody response according to vaccine dose and its associations with clinical outcomes.

## Methods

INVESTED was a randomized, double-blind, active-controlled comparison of high-dose trivalent inactivated influenza vaccine (60 μg of hemagglutinin per strain) or standard-dose quadrivalent inactivated influenza vaccine (15 μg of hemagglutinin per strain) conducted at 157 sites in the United States and Canada over 3 influenza seasons between September 2016 and January 2019.^11^ Eligibility criteria included acute MI within 1 year or HF hospitalization within 2 years of enrollment and at least 1 additional risk factor. The National Heart, Lung, and Blood Institute and ethics committees at each participating site approved the protocol, and each participant provided written informed consent. The trial protocol is shown in Supplement 1.

Antibody titers for influenza antigens were measured at baseline and 4 weeks in patients participating in the immune response substudy during the 2017-2018 and 2018-2019 seasons. Influenza antibody concentrations were measured by hemagglutination inhibition assay performed in duplicate using standard microtiter techniques. Vaccine formulations according to treatment group and season can be found in the eMethods in Supplement 2.

Log-antibody titer differences at baseline and 4 weeks and differences in log-titer change over 4 weeks were examined for the A/H1N1, A/H3N2, and B lineage contained in each year’s influenza vaccine formulations using multivariable linear regression models, adjusted for country. Seroconversion (defined as ≥4-fold titer increase) and seroprotection with titers 1:40 or greater at 4 weeks were compared between vaccine types by logistic regression models, with adjustment for country at baseline. Differences in baseline characteristics according to seroconversion status (for any influenza antigen) in both treatment groups were analyzed by *t* test or χ^2^ test. Predictors of seroconversion for any antigen were assessed using multivariable logistic regression, with adjustment for all baseline characteristics. The associations of seroconversion, seroprotection titer categories, and mean log-titers with the primary composite of first all-cause death or cardiopulmonary hospitalization and key secondary outcomes were assessed by Cox proportional hazards models, using similar models as in the primary analysis.^11^ Statistical analyses were performed using Stata version 17.0 (StataCorp). *P* values less than .05 were considered statistically significant. Data were analyzed from February 2023 to June 2023.

## Results

Among 7154 vaccinations administered to 5260 randomized participants over 3 seasons, antibody data at baseline and 4 weeks were available for 792 participant seasons in 658 participants across 44 sites. In participants with complete antibody data, the mean (SD) age was 66 (11) years, 507 were male (77.1%), and 151 (22.9%) were female. A total of 348 participants were enrolled based on hospitalization for HF (52.9%) and 310 for prior MI (47.1%). Patient characteristics at baseline by randomized treatment and for participants who were and were not included in the immune response substudy are shown in Table 1 and eTable 1 in Supplement 2.

**Table 1.** Baseline Characteristics by Randomized Treatment^a^

Characteristic	No. (%)		*P* value
	Standard-dose quadrivalent vaccine (n = 336)	High-dose trivalent vaccine (n = 322)	
Randomization year			
1	35 (10.4)	33 (10.2)	.98
2	158 (47.0)	154 (47.8)	
3	143 (42.6)	135 (41.9)	
Country			
Canada	148 (44.0)	152 (47.2)	.42
United States	188 (56.0)	170 (52.8)	
Age, mean (SD), y	66.7 (10.9)	65.7 (11.9)	.26
Sex			
Female	85 (25.3)	66 (20.5)	.14
Male	251 (74.7)	256 (79.5)	
Race^b^			
Asian	11 (3.3)	14 (4.3)	.78
Black	23 (6.8)	19 (5.9)	
First Nation and American Indian	0	1 (0.3)	
Other	5 (1.5)	5 (1.6)	
White	297 (88.4)	283 (87.9)	
Ethnicity^c^			
Hispanic and Latino	23 (6.8)	21 (6.5)	.62
Non–Hispanic or Latino	310 (92.3)	300 (93.2)	
Other	3 (0.9)	1 (0.3)	
Ejection fraction, mean (SD), %	44.1 (14.6)	46.8 (15.0)	.04
New York Heart Association functional classification			
1	44 (25.1)	46 (27.1)	.46
2	81 (46.3)	88 (51.8)	
3	45 (25.7)	33 (19.4)	
4	5 (2.9)	3 (1.8)	
BMI, mean (SD)^d^	31.4 (7.2)	30.6 (7.0)	.13
Qualifying event			
Heart failure	178 (53.0)	170 (52.8)	.96
Myocardial infarction	158 (47.0)	152 (47.2)	
Eligibility risk factors^e^			
Age ≥65 y	202 (60.1)	179 (55.6)	.24
Current BMI ≥30	179 (53.3)	151 (46.9)	.10
LVEF <40	131 (39.0)	117 (36.3)	.48
Type 1 or type 2 diabetes	122 (36.3)	107 (33.2)	.41
History of kidney impairment	89 (26.5)	90 (28.0)	.67
Current tobacco smoker	47 (14.0)	50 (15.5)	.58
Prior HF	44 (13.1)	50 (15.5)	.37
Prior MI	71 (21.1)	58 (18.0)	.31
History of ischemic stroke	28 (8.3)	20 (6.2)	.30
History of peripheral artery disease	15 (4.5)	12 (3.7)	.63
Other medical history^f^			
Hypertension	251 (74.7)	239 (74.2)	.89
Dyslipidemia	252 (75.0)	234 (72.7)	.50
Atrial fibrillation	105 (31.2)	102 (31.7)	.91
Coronary artery bypass graft	82 (24.4)	58 (18.0)	.045
Percutaneous coronary intervention	165 (49.1)	152 (47.2)	.63
ICD	50 (14.9)	46 (14.3)	.83
Asthma	41 (12.2)	42 (13.0)	.75
Chronic obstructive pulmonary disease	70 (20.8)	51 (15.8)	.10
Prior influenza vaccine	284 (84.5)	261 (81.1)	.24
Prior influenza infection	94 (28.0)	82 (25.5)	.47
Maintenance medication (index event MI)			
Aspirin	139 (88.0)	134 (88.2)	.96
Stains	148 (93.7)	142 (93.4)	.93
β-Adrenergic blockers	132 (83.5)	128 (84.2)	.87
Maintenance medication (index event HF)			
β-Adrenergic blockers	156 (87.6)	140 (82.4)	.17
Diuretic	150 (84.3)	131 (77.1)	.09
Mineralocorticoid receptor antagonist	61 (34.3)	54 (31.8)	.62
Digoxin	12 (6.7)	17 (10.0)	.27
ACE inhibitor or ARB or ARNI	126 (70.8)	107 (62.9)	.12

Abbreviations: ACE, angiotensin-converting enzyme; ARB, angiotensin II receptor blocker; ARNI, angiotensin receptor neprilysin inhibitor; BMI, body mass index; HF, heart failure; ICD; implantable cardioverter-defibrillator; LVEF, left ventricular ejection fraction; MI, myocardial infarction.

^a^ Analyses were based on the total number of participants. *P* values are reported for differences between patients who were administered high-dose trivalent or standard-dose quadrivalent influenza vaccine.

^b^ Race was collected via participant self-report. The Other category included Native Hawaiian, Pacific Islander, more than 1 race, participant does not want to report, participant does not know, and race not available or missing.

^c^ Ethnicity was collected via participant self-report. The Other category included participant does not want to report, participant does not know, and ethnicity not available or missing.

^d^ Calculated as weight in kilograms divided by height in meters squared.

^e^ Total may be greater than 100% because of multiple risk factors per participant.

^f^ Medical history was collected via patient self-report and medical record review.

Participants who received high-dose vaccine achieved higher titers at 4 weeks for the 3 antigens contained in the trivalent vaccine formulation, compared with those who received standard-dose vaccine (Table 2). Antibody titers for the antigen B/Phuket, which was included only in the quadrivalent standard-dose vaccine, were lower at 4 weeks among those who were administered high-dose vaccine (Table 2). More than 92% of all participants across both groups met seroprotection and higher proportions of participants treated with high-dose vaccine achieved seroconversion (odds ratio, 1.52; 95% CI, 1.12-2.07; *P* = .008) (Table 2). Baseline characteristics by seroconversion status according to randomized treatment are displayed in eTable 2 in Supplement 2. Predictive characteristics of achieving seroconversion for any antigen included absence of prior influenza vaccination, high-dose vaccine, and female sex (eFigure in Supplement 2), though influenza vaccination history did not attenuate the effect of the randomized treatment on seroconversion (*P* = .81 for interaction).

**Table 2.** Mean Log-Titer Change, Seroprotection, and Seroconversion After 4 Weeks by Randomized Treatment^a^

Antigen type	Standard-dose quadrivalent vaccine	High-dose trivalent vaccine	*P* value
**A/H2N3 Hong Kong**			
Baseline mean log-titer (SD)	4.7 (1.6)	4.7 (1.6)	.90
4-wk Mean log-titer (SD)	6.0 (1.3)	6.6 (1.3)	<.001
4-wk Mean change in log-titer (SE)	1.2 (0.1)	1.8 (0.1)	
Difference in log-titer change (95% CI)	0.6 (0.3 to 0.9)		<.001
Seroprotection with titer ≥1:40			
No./total No. (%)	143/149 (96.0)	138/141 (97.9)	
Odds ratio (95% CI)	1.96 (0.48 to 8.00)		.35
Seroconversion			
No./total No. (%)	67/149 (45.0)	78/141 (55.3)	
Odds ratio (95% CI)	1.53 (0.96 to 2.43)		.07
**A/H1N1 Michigan (2017/2018)**			
Baseline mean log-titer (SD)	4.4 (1.4)	4.5 (1.5)	.73
4-wk Mean log-titer (SD)	5.8 (1.3)	6.4 (1.1)	<.001
4-wk Mean change in log-titer (SE)	1.4 (0.1)	1.9 (0.1)	
Difference in log-titer change (95% CI)	0.5 (0.2 to 0.9)		.005
Seroprotection with titer ≥1:40			
No./total No. (%)	145/149 (97.3)	138/141 (97.9)	
Odds ratio (95% CI)	1.23 (0.27 to 5.61)		.79
Seroconversion			
No./total No. (%)	61/149 (40.9)	84/141 (59.6)	
Odds ratio (95% CI)	2.09 (1.30 to 3.35)		.002
**A/H1N1 Michigan (2018/2019)**			
Baseline mean log-titer (SD)	4.2 (1.3)	4.4 (1.4)	.10
4-wk Mean log-titer (SD)	5.5 (1.3)	6.0 (1.3)	<.001
4-wk Mean change in log-titer (SE)	1.3 (0.1)	1.6 (0.1)	
Difference in log-titer change (95% CI)	0.2 (−0.0 to 0.5)		.08
Seroprotection with titer ≥1:40			
No./total No. (%)	238/257 (92.6)	235/245 (95.9)	
Odds ratio (95% CI)	1.87 (0.85 to 4.11)		.12
Seroconversion			
No./total No. (%)	95/257 (37.0)	118/245 (48.2)	
Odds ratio (95% CI)	1.58 (1.11 to 2.26)		.01
**A/H2N3 Singapore**			
Baseline mean log-titer (SD)	4.3 (1.5)	4.3 (1.4)	.99
4-wk Mean log-titer (SD)	5.5 (1.4)	6.0 (1.4)	<.001
4-wk Mean change in log-titer (SE)	1.3 (0.1)	1.8 (0.1)	
Difference in log-titer change (95% CI)	0.5 (0.3 to 0.7)		<.001
Seroprotection with titer ≥1:40			
No./total No. (%)	240/257 (93.4)	239/245 (97.6)	
Odds ratio (95% CI)	2.82 (1.09 to 7.26)		.03
Seroconversion			
No./total No. (%)	104/257 (40.5)	141/245 (57.6)	
Odds ratio (95% CI)	1.99 (1.40 to 2.84)		<.001
**B/Maryland**			
Baseline mean log-titer (SD)	5.1 (1.3)	5.2 (1.3)	.25
4-wk Mean log-titer (SD)	6.3 (1.1)	6.7 (1.2)	<.001
4-wk Mean change in log-titer (SE)	1.2 (0.1)	1.5 (0.1)	
Difference in log-titer change (95% CI)	0.3 (0.0 to 0.5)		.03
Seroprotection with titer ≥1:40			
No./total No. (%)	254/257 (98.8)	242/245 (98.8)	
Odds ratio (95% CI)	0.94 (0.19 to 4.73)		.95
Seroconversion			
No./total No. (%)	98/257 (38.1)	126/245 (51.4)	
Odds ratio (95% CI)	1.72 (1.20 to 2.45)		.003
**B/Brisbane**			
Baseline mean log-titer (SD)	5.4 (1.3)	5.5 (1.5)	.27
4-wk Mean log-titer (SD)	6.5 (1.2)	6.9 (1.1)	.001
4-wk Mean change in log-titer (SE)	1.1 (0.1)	1.4 (0.1)	
Difference in log-titer change (95% CI)	0.3 (−0.0 to 0.6)		.08
Seroprotection with titer ≥1:40			
No./total No. (%)	146/149 (98.0)	139/141 (98.6)	
Odds ratio (95% CI)	1.51 (0.25 to 9.22)		.66
Seroconversion			
No./total No. (%)	49/149 (32.9)	63/141 (44.7)	
Odds ratio (95% CI)	1.60 (0.98 to 2.59)		.06
**B/Phuket**			
Baseline mean log-titer (SD)	5.1 (1.4)	5.0 (1.4)	.25
4-wk Mean log-titer (SD)	6.3 (1.1)	5.7 (1.3)	<.001
4-wk Mean change in log-titer (SE)	1.2 (0.1)	0.7 (0.1)	
Difference in log-titer change (95% CI)	−0.5 (−0.6 to −0.3)		<.001
Seroprotection with titer ≥1:40			
No./total No. (%)	397/406 (97.8)	366/386 (94.8)	
Odds ratio (95% CI)	0.42 (0.19 to 0.93)		.03
Seroconversion			
No./total No. (%)	156/406 (38.4)	89/386 (23.1)	
Odds ratio (95% CI)	0.47 (0.35 to 0.65)		<.001
**Any antigen**			
Seroprotection with titer ≥1:40			
No./total No. (%)	406/406 (100)	386/386 (100)	
Odds ratio (95% CI)	NA		NA
Seroconversion			
No./total No. (%)	267/406 (65.8)	288/386 (74.6)	
Odds ratio (95% CI)	1.52 (1.12 to 2.07)		.008

Abbreviation: NA, not applicable.

^a^ Log-titer differences at baseline and 4 weeks and differences in log-titer change over 4 weeks were examined using multivariable linear regression models, adjusted for country. Seroconversion was defined as a ≥4-fold titer increase between baseline and 4 weeks for any of the antigens A/H2N3 Hong Kong, A/H1N1 Michigan, A/H2N3 Singapore, B/Maryland, B/Brisbane, or B/Phuket. Estimated odds ratios and 95% CIs were derived from logistic regression models, adjusted for country. All analyses were based on the total number of participant-seasons. *P* values are reported for differences between patients who were administered high-dose trivalent or standard-dose quadrivalent influenza vaccine.

The risk for the primary outcome did not significantly differ between participants who did and those who did not achieve seroconversion (hazard ratio [HR], 1.09; 95% CI, 0.79-1.53; *P* = .59), with similar findings for key secondary outcomes (Figure and eTable 4 in Supplement 2). In addition, there were no associations between risk for the primary outcome and ordinal levels of seroprotection titers for any antigen (HR, 1.06; 95% CI, 0.74-1.54; *P* = .74) and continuously modeled mean log titers (eTables 5 and 6 in Supplement 2). The association between seroconversion and the primary outcome was not modified by randomized treatment (*P* = .38 for interaction).

**Figure.**
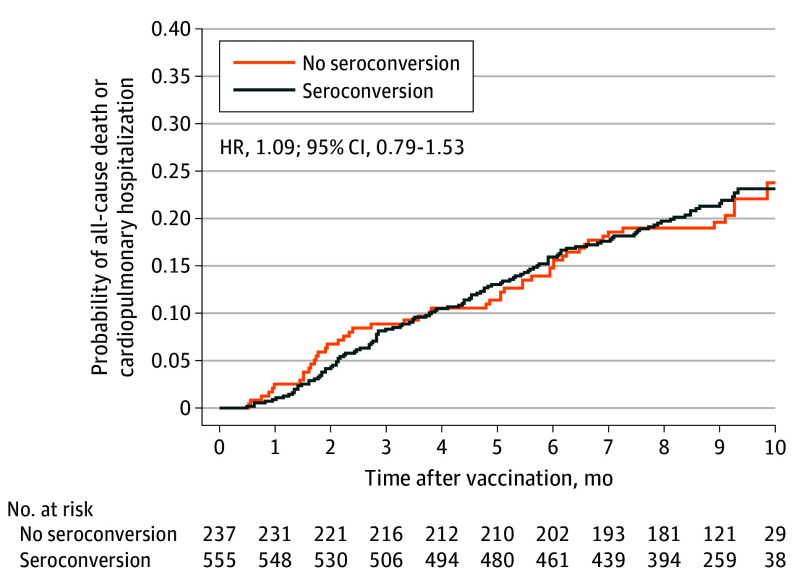
Cardiopulmonary Hospitalization or All-Cause Death by Seroconversion Status Seroconversion was defined as a 4-fold or greater titer increase between baseline and 4 weeks for any of the antigens A/H2N3 Hong Kong, A/H1N1 Michigan, A/H2N3 Singapore, B/Maryland, B/Brisbane, or B/Phuket. Analyses were based on each participant season, with the number of all participant-seasons combined. HR indicates hazard ratio.

## Discussion

In this prespecified analysis of the INVESTED trial, high-dose vaccine resulted in a greater increase in antibody titers and higher seroconversion rates in patients with HF or prior MI. The magnitude of antibody response to influenza vaccine was not associated with the risk for all-cause mortality or cardiopulmonary hospitalizations.

The more pronounced antibody response observed in participants who received high-dose vaccine confirmed expected patterns by randomization, with greater increases in mean titer and seroconversion rates for all antigens included in the high-dose vaccine formulation, together with the lower response for the B-lineage antigen contained only in the standard-dose quadrivalent vaccine. Consistent with observations from previous vaccine trials, the absence of a prior influenza vaccination was associated with higher seroconversion rates, which may be attributable to lower preexisting antibody titers.^12^ However, given the similar prevalence of prior influenza vaccination at baseline in both treatment groups, with no observed attenuation of the effect of the prior vaccination on seroconversion, vaccination history is unlikely to explain the neutral primary result of INVESTED.^11^ While most comorbidities did not influence the likelihood of seroconversion, seroconversion rates may be lower among individuals with many risk factors, adding to previous suggestions of potentially less vigorous humoral response in multimorbidity.^10^

Although high-dose vaccine elicited a more robust immune response, higher seroprotection levels and seroconversion status did not translate into benefits in all-cause mortality or cardiopulmonary hospitalizations in INVESTED, regardless of treatment group. While high-dose compared with standard-dose vaccine has been shown to provide greater protection against influenza infections and associated complications, these events were not specifically examined in INVESTED.^7,11^ Despite the higher prevalence of seroconversion with high-dose vaccine, it is conceivable that the high proportions of seroprotection achieved in both groups may have contributed to the observed similar rates of primary events.^13^ Likewise, the potentially attenuated humoral response among patients with high comorbidity burden and the elevated underlying risk of clinical events in the study population may have influenced the association between achieved antibody titers and clinical outcomes.^8,10^ Moreover, vaccine effects on atherothrombotic events may be in part attributable to pleiotropic anti-inflammatory properties that may not correlate with the humoral response.^14^ These results indicate that the previously reported neutral effects on adverse clinical events in INVESTED are likely not attributed to a lack of antibody response with high-dose compared with standard-dose vaccines. Accordingly, the magnitude of humoral response appears to be of limited predictive value for vaccine efficacy on adverse cardiopulmonary outcomes in patients at high cardiovascular risk, with seroprotection levels achieved with standard-dose compared with high-dose vaccine potentially having similar protective effects. The ongoing large-scale, pragmatic, registry-based DANFLU-2 (A Pragmatic Randomized Trial to Evaluate the Effectiveness of High-Dose Quadrivalent Influenza Vaccine vs Standard-Dose Quadrivalent Influenza Vaccine in Older Adults) involving older adults may complement current evidence on the efficacy of quadrivalent high-dose vs standard-dose influenza vaccine on adverse clinical events, including cardiopulmonary outcomes.^15^

### Limitations

Although the immune response substudy included more than 10% of all INVESTED participants randomized across geographically diverse regions of the United States and Canada, the smaller sample size and differences in baseline characteristics may affect the generalizability of the results. Moreover, missing data may have influenced the analyses, although the distribution of missing antibody titers was similar across both treatment groups. While antibody titers were determined at baseline and after 4 weeks, long-term effects on humoral response could not be assessed. In addition, INVESTED did not have a nonvaccinated placebo arm, preventing determining whether receipt of any influenza vaccine reduces the risk for adverse clinical events compared with no vaccination. Finally, the findings preclude conclusions on other vaccine formulations.

## Conclusions

This analysis of the INVESTED trial found that in patients with HF or prior MI, high-dose vaccine elicited a more robust humoral response, compared with standard-dose vaccine, with no association between seroconversion status and the risk for cardiopulmonary hospitalizations or all-cause mortality. Vaccination remains critical for the prevention of influenza in high-risk populations.

## Notes

Supplement 1. Trial protocol

Supplement 2. **eTable 1.** Baseline Characteristics by Immune Response Sub-Study Participation Status **eTable 2.** Baseline Characteristics According to Seroconversion Status by Randomized Treatment **eTable 3.** Seroconversion by Total Number of Eligibility Risk Factors **eTable 4.** Primary and Key Secondary Outcomes by Seroconversion Status **eTable 5.** Cardiopulmonary Hospitalization or All-Cause Death According to Ordinate Levels of Seroprotection after 4-Weeks **eTable 6.** Cardiopulmonary Hospitalization or All-Cause Death According to Mean Log-Titer Levels at 4 Weeks as a Continuous Measure **eFigure.** Multivariate Predictors of Seroconversion Status **eMethods.** Vaccine Formulations by Randomized Treatment According to Influenza Season

Supplement 3. Data sharing statement
