# Supplementary material for: Influenza Vaccine Immune Response in Patients With High-Risk Cardiovascular Disease: A Secondary Analysis of the INVESTED Randomized Clinical Trial
Source: JAMA Cardiol. 2024 Apr 7;9(6):574–81. doi: 10.1001/jamacardio.2024.0468 (PMC11000133; doi:10.1001/jamacardio.2024.0468)
Supplement: Supplement 1. — Trial protocol [file jamacardiol-e240468-s001.pdf]

INfluenza Vaccine to Effectively Stop Cardio Thoracic Events and Decompensated heart failure (INVESTED)

Protocol

VERSION 4.0

DATE: 23 July2019

This document is confidential.
No part of it may be transmitted, reproduced, published, or used by other persons without prior written authorization from the Sponsor or Principal Investigator.

Funding Agency	
National Heart, Lung, and Blood Institute (NHLBI),<br>National Institutes of Health (NIH)<br>Lawton Cooper, MD, MPH<br>Medical Officer, Division of Prevention and Population Sciences<br>cooperls@nhlbi.nih.gov<br>301-435-0419	Sanofi Pasteur<br>1 Discovery Drive<br>Swiftwater, PA 18370<br>570-839-7187
**Clinical Coordinating Center**	
Orly Vardeny, PharmD, MS, Co-Principal Investigator<br>Associate Professor of Medicine<br>University of Minnesota Medical School<br>1 Veterans Drive<br>Minneapolis, MN 55417<br>ovardeny@umn.edu<br>612-467-4586	Scott Solomon, MD, Co-Principal Investigator<br>Professor, Harvard Medical School<br>Brigham and Women's Hospital<br>75 Francis Street<br>Boston, MA 02115<br>ssolomon@rics.bwh.harvard.edu<br>857-307-1954
**Data Coordinating Center**	
KyungMann Kim, PhD, DCC Principal Investigator<br>Professor, Biostatistics and Medical Informatics<br>School of Medicine and Public Health<br>University of Wisconsin-Madison<br>K6/438 Clinical Science Center<br>600 Highland Ave<br>Madison, WI 53792-4675<br>kyungmann.kim@wisc.edu<br>608-265-6380	Ken Wood, MS<br>Project Manager, Data Management<br>Frontier Science and Technology Research Foundation, Inc.<br>4033 Maple Road<br>Amherst, NY 14226<br>wood@fstrf.org<br>716-834-0900 x7235
**Canadian Consortium**	
Jacob Udell, MD, MPH<br>Assistant Professor of Medicine<br>University of Toronto<br>76 Grenville St<br>Toronto, Ontario, M5S-1B1<br>jay.udell@utoronto.ca<br>416-351-3732 x2459	Michael Farkouh, MD, MSc<br>Professor of Medicine<br>Peter Munk Cardiac Centre, University Health Network<br>585 University Avenue, Room 4N474<br>Toronto, Ontario M5G 2N2<br>michael.farkouh@uhn.ca<br>416-340-3141
**VA Consortium**	
J. Michael Gaziano, MD, MPH<br>Professor, Harvard Medical School<br>Chief, Division of Aging, Brigham and Women's Hospital<br>jmgaziano@partners.org<br>Michael.Gaziano@va.gov<br>617-278-0848	Jacob Joseph, MD, MB, BS<br>Associate Professor of Medicine<br>Brigham and Women's Hospital &<br>Cardiology Section, VA Boston Healthcare<br>Jacob.joseph@va.gov<br>857-203-6841
**PCORnet**	
Adrian Hernandez, MD, MHS<br>Professor of Medicine,<br>Duke University School of Medicine<br>Duke Box 31259, Durham, NC 27710<br>adrian.hernandez@duke.edu<br>919-668-7515	
**Serology Laboratories**	

Brigham and Women's Hospital<br>75 Francis Street<br>Boston, MA 02115	Peter Munk Cardiac Centre Clinical Trials Biomarker Laboratory<br>200 Elizabeth Street, MBRC3R414-21<br>Toronto, Ontario M5G 1L7
**Study Vaccine Distribution**	
Biologics, Inc.<br>120 Weston Oaks Court<br>Cary, North Carolina 27513	Elliott Lee<br>Clinical Research Services<br>Elliott.lee@mckesson.com<br>919.459.4949

TABLE OF CONTENTS

1.0 LIST OF ABBREVIATIONS 7

2.0 KEY ROLES AND CONTACT INFORMATION 8

2.1 Clinical Coordinating Center (CCC) 8
2.2 Data Coordinating Center (DCC) 8
2.3 Executive Committee 8
2.4 Steering Committee 8
2.5 Clinical Endpoints Committee 8
2.6 Clinical Site Networks 8
2.7 Site Management 9

3.0 STUDY DESIGN 9

4.0 STUDY SUMMARY 10

5.0 BACKGROUND AND RATIONALE 11

5.1 Rationale 11
5.2 Description of Investigational Product 12
5.3 Summary of Findings from Relevant Nonclinical Studies and Clinical Trials 12
5.4 Summary of the Known and Potential Risks and Benefits to Human Subjects 12
5.5 Correlative Studies 13

6.0 TRIAL OBJECTIVES 13

6.1 Primary Objective 13
6.2 Secondary Objectives 13
6.3 Correlative Study Objectives 14
6.4 Exploratory Objectives 14

7.0 STUDY POPULATION 15

7.1 Accrual Goal 16
7.2 Recruitment Plan 16

8.0 INVESTIGATIONAL PRODUCT 16

8.1 Fluzone® Quadrivalent Influenza Vaccine (15 µg/strain, QIV) 16
8.2 Fluzone® High-Dose Trivalent Influenza Vaccine (60 µg/strain, TIV) 17
8.3 Properties 17
8.4 Acquisition & Shipping 17
8.5 Product Storage and Stability 17
8.6 Accountability Procedures 17
8.7 Dosage and Administration 18
8.8 Adverse Reactions 19
8.9 Duration of Therapy 19
8.10 Duration of Follow-Up 19
8.11 Medications/Treatments Permitted and Not Permitted during the Trial 19
8.12 Removal of Subjects from Protocol Therapy 20
8.13 Duration of Therapy 20

9.0 STUDY PROCEDURES 21

9.1 Screening/Baseline Visit 21
9.2 Follow-up 22
9.3 Correlative Study Visit (immune response sub-study, for a subset of subjects only, after the Vanguard year) 23
9.4 Schedule of Time and Events Table 23
9.5 Subjects who decline to continue receiving influenza vaccine ..... **Error! Bookmark not defined.**
9.6 Subjects who withdraw consent ..... **Error! Bookmark not defined.**

10.0 OUTCOME MEASURES 24
10.1 Primary Efficacy Endpoint 24
10.2 Secondary Efficacy Endpoints 24
10.3 Exploratory Efficacy Endpoints 24
10.4 Primary Safety Endpoint 24
10.5 Secondary Safety Endpoints 24
10.6 Correlative study endpoints (up to 3,000 subjects) 24
11.0 ASSESSMENT OF SAFETY 25
11.1 Specification of Safety Parameters 25
11.2 Classification of an Adverse Event 25
11.3 Time Period and Frequency for Event Assessment and Follow-Up 26
11.4 Reporting Procedures 27
11.5 Study Termination Rules 28
11.6 Unblinding Procedure 28
11.7 Safety Oversight 28
12.0 CORRELATIVE (IMMUNE RESPONSE, BIOMARKER AND DNA) STUDIES 28
12.1 Sample Collection Guidelines 29
12.2 Assay Methodology for immune response studies 29
12.3 Specimen Banking 29
13.0 STATISTICAL CONSIDERATIONS 30
13.1 Statistical Analysis Plan 30
13.2 Analysis Population 30
13.3 Analysis of Baseline Characteristics 30
13.4 Analysis of Primary Endpoints 31
13.5 Analysis of Secondary Endpoints 31
13.6 Analysis of Safety Endpoints. 32
13.7 Analysis of Immune Outcomes (Correlative Study Endpoints) 32
13.8 Additional Analyses 32
13.9 Subgroup Analysis 32
13.10 Missing data 33
13.11 Interim Analysis 33
13.12 Number of Subjects to be Enrolled 33
13.13 Level of Significance 34
13.14 Stopping Rules for Termination of the Trial 34
13.15 Spurious Data Procedures 34
13.16 Deviation Reporting Procedures 35
13.17 Subjects to be Included in Analyses 35
13.18 Measures to Minimize/Avoid Bias 35
13.19 Data and Safety Monitoring Board (DSMB) 36
13.20 Clinical Endpoints Committee 36
14.0 ETHICS AND PROTECTION OF HUMAN SUBJECTS 36
14.1 Ethical Standard 36
14.2 Institutional Review Board (IRB) 37
14.3 Informed Consent Process 37
14.4 Exclusion of Women, Minorities and Children (Special Populations) 37
15.0 TRIAL MANAGMENT AND COMMUNICATION PLAN 37
15.1 Monitoring IRB Approvals 37
15.2 Monitoring of Modifications 38
15.3 Problem Management 38
15.4 Communication 38
16.0 DATA COLLECTION AND QUALITY ASSURANCE 38

16.1 Data Management Responsibilities 38
16.2 Clinical Trial Data Management System 39
16.3 Data Management Procedures 39
16.4 Direct Access to Source Data 40
16.5 Specimen Collection Management 40
16.6 Confidentiality of Data 40
16.7 Confidentiality of Subject Records 40
16.8 Protocol Deviations 41

17.0 PUBLICATION POLICY 41

18.0 REFERENCES 41

APPENDIX A 45

APPENDIX B 46

APPENDIX C 49

1.0 LIST OF ABBREVIATIONS

Abbreviation	Abbreviation Term
ACIP	Advisory Committee on Immunization Practices
ADL	Activities of Daily Living
AMI	Acute Myocardial Infarction
AE	Adverse Event
CCC	Clinical Coordinating Center
CCS	Canadian Cardiovascular Society
CDC	Centers for Disease Control and Prevention
CEC	Clinical Endpoints Committee
CI	Confidence Interval
CRF	Case Report Form
CSR	Clinical Study Report
CTDMS	Clinical Trial Data Management System
CV	Cardiovascular
CVD	Cardiovascular Disease
DCC	Data Coordinating Center
DSMB	Data and Safety Monitoring Board
EC	Executive Committee
eCRF	Electronic Case Report Form
EDC	Electronic Data Capture
FDA	Food and Drug Administration
GBS	Guillain-Barre syndrome
GCP	Good Clinical Practice
HAU	Hemagglutinin Units
HIA	Hemagglutination Inhibition Assay
HIPAA	Health Insurance Portability and Accountability Act
HF	Heart Failure
IP	Investigational Product
ICF	Informed Consent Form
ICH	International Conference on Harmonization
IM	Intramuscular
IRB	Institutional Review Board
IQR	Interquartile Range
ITT	Intention-to-Treat
LVEF	Left ventricular ejection fraction
MACE	Major Adverse Cardiovascular Events
MedDRA	Medical Dictionary for Drug Regulatory Activities
MI	Myocardial Infarction
mITT	Modified Intention-to-Treat
MOP	Manual of Procedures
NYHA FC	New York Heart Association Functional Class
NIH	National Institutes of Health
NHLBI	National Heart, Lung, and Blood Institute
PHI	Personal Health Information
PHIPA	Personal Health Information Privacy and Access Act
PI	Principal Investigator
QA	Quality Assurance
QC	Quality Control
QIV	Quadrivalent influenza vaccine
RCT	Randomized Controlled Trial
SAE	Serious Adverse Event
SAP	Statistical Analysis Plan
SC	Steering Committee
SOP	Standard Operating Procedure
StaRRS	Statistical Registration and Randomization System

SUSAR	Suspected Unexpected Serious Adverse Reaction
TIV	Trivalent Influenza Vaccine

2.0 KEY ROLES AND CONTACT INFORMATION

2.1 Clinical Coordinating Center (CCC)

The CCC will be responsible for overall study execution, including protocol development, identification and recruitment of coordinating networks/clinical sites and Principal Investigators (PIs), comprehensive site training (including initial protocol and subsequent modifications), vaccine blinding and delivery, medical monitoring, handling of subject-related issues that may arise, coordination of clinical event ascertainment efforts, and interfacing with the Data Coordinating Center.

2.2 Data Coordinating Center (DCC)

The DCC will be responsible for providing expertise and support for data management, quality control and quality assurance, information technology for communication and trial conduct monitoring, and statistical methods for design including randomization, interim monitoring, analysis both interim and final, interpretation of findings from analysis, preparation of results in tabular and graphical formats for presentation and publication of findings from the trial.

2.3 Executive Committee

The Executive Committee will consist of the CCC and DCC principal investigators and each of the consortium PIs, in addition to influenza vaccine experts, and will be responsible for overall operational aspects of the trial.

2.4 Steering Committee

The Steering Committee will consist of the Executive Committee plus additional members. The larger steering committee will add specific expertise, especially in infectious disease, vaccine trials, clinical trials, and public policy. The Steering Committee will be consulted on all protocol decisions and on all major operational aspects of the trial.

2.5 Clinical Endpoints Committee

The Clinical Endpoints Committee will categorize/classify all potential study clinical endpoints in a blinded fashion according to pre-specified guidelines. The committee will be comprised of a chair and two physician reviewers with expertise in endpoint categorization/classification. The primary trial endpoint is a composite of all-cause death or cardiopulmonary hospitalization. Recognizing that limited source material may be available, the committee will receive information about all hospitalizations and determine whether the primary reason for the hospitalization was either cardiac or pulmonary.

2.6 Clinical Site Networks

2.6.1 Pan-Canadian Network

University of Toronto, this network will consist of approximately 60 sites from geographically diverse regions in Canada that have a combined accrual goal of approximately 4,000 subjects.

2.6.2 Brigham and Women's Hospital (BWH) Consortium

This network will oversee four Clinical Data Research networks that are members of the National Patient-Centered Clinical Research network (PCORnet) and other individual sites, including those with an established track record for recruiting underrepresented minorities. The BWH Consortium has a combined accrual goal of 2,800 subjects.

2.6.3 The Veterans Administration Consortium

Headquartered at the Boston VA Network Coordinating Center, this network will consist of approximately 35 sites that have a combined accrual goal of 2,500 subjects.

2.7 Site Management

The CCC will maintain records of sites' clinical research essential documents. These records will be located in the INVESTED web Trial Master File maintained by sites with the CCC oversight.

The DCC will monitor and identify issues related to data entry and quality control and assurance. A risk-based monitoring strategy will be employed. Sites in which problems are identified will be visited by leadership from their consortium as necessary and sites that do not conform to good clinical practices will be closed.

Recruitment will be monitored in real-time by the DCC. Recruitment reports will be run weekly and provided to the Executive Committee for review and posted on the INVESTED web portal as part of the DCC's trial conduct monitoring activities.

3.0 STUDY DESIGN

INVESTED is a randomized, double-blind, active-controlled, multi-site trial comparing high-dose (60 µg per vaccine viral strain) trivalent influenza vaccine (TIV) to standard-dose (15 µg per viral strain) quadrivalent influenza vaccination (QIV) for up to three influenza seasons in 9,300 high-risk cardiovascular disease patients with a history of myocardial infarction in the previous 12 months OR history of heart failure hospitalization in the previous 24 months (Figure 1). Subjects will be randomized in a 1:1 ratio to high-dose or standard-dose, using permuted blocks of random block size, balanced by site, without stratification, except for the natural stratification by influenza season.

The total trial duration is 4 years including site initiation, a Vanguard season followed by 3 influenza seasons, and follow-up until the end of the last influenza recruiting season. During the Vanguard phase (2016/2017 influenza season), up to 500 subjects will be enrolled from a subset of sites.

Figure 1: Study Schema

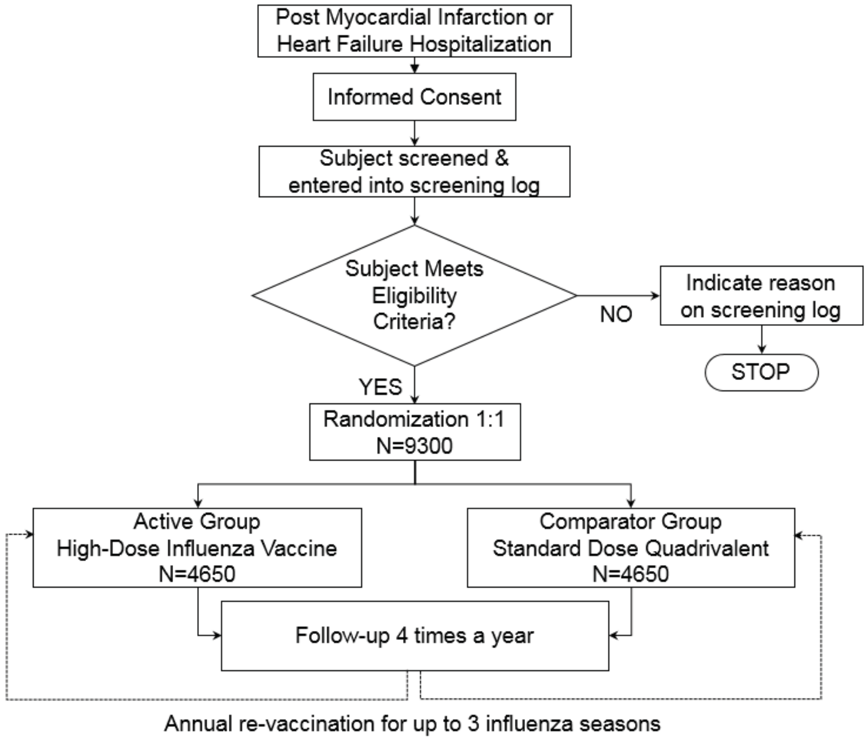

4.0 STUDY SUMMARY

Title	INfluenza Vaccine to Effectively Stop CardioThoracic Events and Decompensated heart failure (INVESTED)
Short Title	Influenza vaccine in cardiovascular disease
Phase	Phase IV
Methodology	This is a multicenter, prospective, randomized, double-blind, active-controlled trial comparing two doses of influenza vaccine in high risk cardiovascular patients (history of myocardial infarction or heart failure).
Study Duration	Approximately four years (Fall 2016 – Summer 2020)
Study Center(s)	Approximately 180 clinical sites in the United States and Canada
Objective(s)	The primary objective is to compare high-dose influenza vaccine to standard-dose vaccine in terms of the time to first occurrence of death or cardiopulmonary hospitalization within each enrolling season.
Number of Subjects	9,300
Funding Source	NHLBI U01HL130163 and U01HL130204 and Sanofi-Pasteur
Clinicaltrials.gov	NCT02787044
FDA Status	Both study drugs are FDA-approved for prevention of seasonal influenza disease caused by influenza A subtype viruses and type B virus. Use of the high dose trivalent vaccine is investigational in adults under 65 years old. The FDA has deemed this study IND Exempt.
Health Canada Status	Both study drugs are Health Canada-approved for prevention of seasonal influenza disease caused by influenza A subtype viruses and type B virus. Use of the high dose trivalent vaccine is investigational in adults under 65 years old. This study is regulated by Health Canada for the participating Canadian sites.
Diagnosis and Main Inclusion Criteria	Recent hospitalization for acute myocardial infarction (AMI) or heart failure (HF)

Study Product(s), Dose, Route, Regimen	Fluzone® High-Dose Trivalent Influenza Vaccine (60 µg/strain, TIV<br>1 dose (0.5 mL) IM annually
Reference therapy	Fluzone® Quadrivalent Influenza Vaccine (15 µg/strain, QIV)<br>1 dose (0.5 mL) IM annually
Duration of administration	1 dose annually for up to three influenza seasons
Statistical Methodology	A modified intention-to-treat (mITT) analysis comparing time to first occurrence of death or cardiopulmonary hospitalization within each enrolling season.

5.0 BACKGROUND AND RATIONALE

5.1 Rationale

Influenza infection is known to be associated with increased risk of cardiovascular (CV) events.[1-3] Several lines of investigation suggest that influenza vaccine might attenuate cardiac risk in high-risk populations.[4-8] However, influenza vaccine is widely and profoundly underutilized in this population.[9-11] Our meta-analysis of randomized controlled trials (RCTs) suggests the use of influenza vaccine compared with placebo or routine care in high-risk patients is associated with a lower risk of major adverse cardiovascular events (MACE) (2.9% vs. 4.7%; risk ratio [RR] 0.64, 95% CI, 0.48-0.86; P=0.003).[12] Five key observations from this analysis and our mechanistic work form the rationale to proceed with a definitive randomized, controlled trial to extend these findings: (1) a treatment interaction detected between patients with (RR 0.45, 95% CI, 0.32-0.63) and without (RR 0.94, 95% CI, 0.55-1.61) a recent acute coronary syndrome (P-interaction=0.02) suggests that higher risk cardiovascular patients derive the greatest cardioprotective benefit from influenza vaccine; (2) further reduction in MACE was suggested in RCTs of more potent versus standard influenza vaccine (RR 0.72, 95% CI, 0.46-1.13); (3) patients with advanced heart disease in the form of heart failure (HF) who contract influenza are at significantly greater risk for acute HF and CV hospitalizations during winter months compared with similar high-risk patients without influenza infection; (4) evidence from our lab and others indicates that patients with HF have reduced influenza antibody titers and altered cytokine production following influenza vaccination (immunosenescence), and that this reduction is related to the severity of underlying heart disease;[13-15] and (5) our pilot RCT suggests that immunosenescence in patients with advanced heart disease may be overcome with higher-dose influenza vaccine.[16]

Several studies in older adults have tested higher vaccine doses and have shown higher antibody titer production compared to a standard vaccine dose, with no increase in serious adverse events.[17-19] Recently, a phase 3 trial of 32,000 healthy elderly adults demonstrated a reduced rate of laboratory-confirmed influenza among subjects randomized to Fluzone High-Dose compared with standard Fluzone with no increase in serious adverse events. An analysis from this trial showed that the high-dose vaccine tended to reduce the risk of pneumonia, all-cause hospitalizations, and cardiopulmonary events.[20] Though the high-dose vaccine is currently licensed for use in medically stable individuals over age 65 years in the United States and Canada, the Advisory Committee on Immunization Practices (ACIP) does not advocate use of high-dose over standard-dose in older adults. Our pilot randomized controlled trial in high-risk heart failure patients suggests that high-dose vaccine can confer significantly increased immune response than standard-dose vaccine in this vulnerable population. However, high-dose vaccine has not been specifically studied in high-risk cardiac patients in relation to cardiopulmonary outcomes.

INVESTED is a large, adequately powered, multicenter trial to assess the cardiopulmonary benefit of high- compared with standard-dose influenza vaccine in a high-risk cardiovascular population. In contrast to more complex interventions in patients with cardiac disease, the major potential benefits of this intervention are a) the ease of administration, b) low cost, c) 100% vaccination adherence and d) well established and extremely low risk. This trial, if positive, has the potential to

substantially impact a major population attributable CV risk, change practice, and inform health policy by boosting utilization of influenza vaccination.

5.2 Description of Investigational Product

Subjects will be assigned to receive one of two formulations of influenza vaccine: high-dose (Fluzone® high-dose TIV), or standard-dose (Fluzone® QIV). Vaccine will be administered intramuscularly once at randomization and yearly thereafter. Study medication will be provided by Sanofi-Pasteur.

5.3 Summary of Findings from Relevant Studies and Clinical Trials

DiazGranados et al. conducted a phase IIIb–IV, multicenter, randomized, double-blind, active-controlled trial to compare high-dose influenza vaccine (60 µg HAU per strain) with standard-dose trivalent, inactivated influenza vaccine (15 µg HAU per strain) in 31,989 adults 65 years of age or older. The primary endpoint of the study was the occurrence, at least 14 days after vaccination, of laboratory-confirmed influenza caused by any influenza viral types or subtypes, in association with a protocol-defined influenza-like illness. In total, 228 subjects in the high-dose group (1.4%) and 301 subjects in the standard-dose group (1.9%) had laboratory confirmed influenza caused by any viral type or subtype associated with a protocol-defined influenza-like illness (relative efficacy, 24.2%; 95% confidence interval [CI], 9.7 to 36.5%). Antibody titers were significantly higher among those who received high-dose versus standard-dose vaccine.

Keitel et al. compared several TIV doses (15, 30, and 60 µg HAU) in 202 adults over 65 years of age in a randomized, controlled trial.[17] Antibody titers were measured in serum by hemagglutination inhibition assay (HIA), the gold standard for measurement of humoral immune responses. Safety endpoints included the frequency and severity of local and systemic adverse reactions. There were dose-related increases in mean antibody titers and rates of seroprotection for the A/H3N2 and B-type antigens. Highest titers were achieved with the 60 µg dose. These findings were confirmed in a phase II randomized study comparing 60 µg to 15 µg TIV in 414 older adults.[21] Mean antibody titers increased significantly after immunization for both groups, but the higher dose induced a significantly greater increase in titers for all three vaccine antigens ($p<0.01$ for comparison of high- vs. standard-dose TIV for A/H1N1 and A/H3N2, and $p=0.04$ for B-type).

5.4 Summary of the Known and Potential Risks and Benefits to Human Subjects

Mild soreness at the injection site, headache, and muscle aches are the most commonly reported symptoms following influenza vaccination. As with all vaccines, recipients of the influenza vaccine incur a slight risk of experiencing a severe allergic reaction. Signs of serious allergic reaction can include breathing problems, hoarseness or wheezing, hives, paleness, weakness, a fast heartbeat, or dizziness. If reactions like this occur, it is within a few minutes to a few hours after the vaccine. There is some evidence of a link between some influenza vaccines and Guillain-Barré syndrome (GBS). GBS is a disease that can cause serious and even permanent damage to the nervous system. About 1 in 100,000 people per year develop GBS, a small number of these cases may be triggered by influenza vaccines.

The safety of higher doses of influenza vaccine has been evaluated in three studies in older adults.[17,18,21] Couch et al. compared standard-dose (15 µg) to high-dose (60µg) vaccine in a multi-site, phase II randomized, double-blind, stratified (based on prior vaccine use) study. Subjects were instructed to record their temperature and adverse effects on a daily basis for 12 days. Each subject was contacted by phone between days 8 and 12 and at 7 months to review occurrence of adverse events. Investigators found more reports of local pain and myalgias related to high-dose vaccine. There were no significant differences in serious adverse events (allergic reactions, sustained myalgias) between the groups.

Keitel et al. compared safety and efficacy of various influenza vaccine doses in patients older than 65 years. Subjects were randomized to receive either placebo, 15, 30, or 60 µg of influenza vaccine. Oral temperature, injection site, and systemic symptoms and signs were recorded in a daily diary for 7 days post immunization. Subjects were assessed at 30 minutes, 2 and 28 days for serious adverse events. No severe injection site discomfort was reported as most reactions were mild. Dose-related increases in injection site discomfort and redness or swelling were observed but all dosages were well tolerated. Systemic symptoms (headache, malaise, nausea, body aches) did not differ between groups. Alternative to this study, subjects may receive influenza vaccine as per standard of care.

5.5 Rationale for the study

As noted in Sections 5.3 and 5.4 above, the risk/benefit ratio of this study is favorable. The risk of adverse vaccine reactions is low, and most are relatively minor in nature. There will be some unavoidable inconvenience to subjects and families because of study visits and optional blood draws. Subjects are not expected to benefit directly from participating in this study. It is possible that subjects who receive a higher dose of vaccine will have a stronger immune response, which may protect them more against influenza virus infection. Regardless of treatment assignment, subjects will receive influenza vaccination as part of the study, which has shown to be beneficial in reducing the chance of getting influenza virus infections.

All study costs not related to standard clinical care and performed solely for the purpose of research will be paid for by the study funding agency. Given the anticipated benefits to subjects and others with influenza vaccine, the risks are reasonable.

5.6 Correlative Studies

5.6.1 Immune Responses Substudy
Antibody titers to influenza vaccine antigens, seroprotection, and seroconversion will be assessed in a subgroup of subjects (N=up to 3,000) to test the hypothesis that a higher influenza vaccine dose will result in more pronounced humoral immune response, evidenced by greater mean titers post-vaccination and higher antibody titer changes from baseline, and to test the hypothesis that higher antibody concentrations will be associated with a reduced rate of the composite of all-cause death and cardiopulmonary hospitalization

5.6.2 Adult Congenital Heart Disease (ACHD) Substudy
A 500-patient cohort comprised of adults with congenital heart disease (ACHD) will be assessed to test the hypothesis that high dose vaccine will reduce the composite of death or cardiopulmonary hospitalizations. See Appendix B.

6.0 TRIAL OBJECTIVES

6.1 Primary Objective

The primary objective is to compare high-dose trivalent influenza vaccine to standard-dose quadrivalent influenza vaccine on time to first occurrence of death or cardiopulmonary hospitalization within each enrolling season.

6.2 Secondary Objectives

6.2.1 To compare the effect of high-dose influenza vaccine versus standard-dose vaccine on total (first and recurrent) cardiopulmonary hospitalizations or death

6.2.2 To compare the effect of high-dose influenza vaccine versus standard-dose vaccine on time to first occurrence of cardiovascular death or cardiovascular hospitalization within each enrolling season

6.2.3 To compare the effect of high-dose influenza vaccine versus standard-dose vaccine on time to first occurrence of death or cardiopulmonary hospitalization across all enrolling seasons

6.2.4 To compare the effect of high-dose influenza vaccine versus standard-dose vaccine on the individual components of the primary endpoint

6.2.5 To compare the safety and tolerability of high-dose influenza vaccine versus standard-dose vaccine in a high-risk cardiovascular population

6.3 Correlative Study Objectives

6.3.1 Immune Response Substudy

To test the hypothesis that a higher influenza vaccine dose will result in more pronounced humoral immune response, evidenced by greater mean titers post-vaccination and higher antibody titer changes from baseline, and to test the hypothesis that higher antibody concentrations will be associated with a reduced rate of the composite of all-cause death and cardiopulmonary hospitalization. Antibody titers to influenza vaccine antigens, seroprotection, and seroconversion will be assessed in a subgroup of subjects (N=up to 3,000). Biospecimens will also be utilized to measure genetic markers and other biomarker levels.

6.3.2 ACHD Substudy

The primary objective is to test the hypothesis that high dose (4x) trivalent influenza vaccine will reduce the composite of death or cardiopulmonary hospitalizations compared with standard dose quadrivalent influenza vaccine in the ACHD subgroup of the INVESTED trial.

The primary endpoint is the time to first occurrence of death or cardiopulmonary hospitalization within each influenza season in the ACHD population.

Secondary objectives are:

6.3.2.1 To test the hypothesis that compared to non-ACHD patients enrolled in the INVESTED trial, ACHD patients will have a higher composite endpoint of death or cardiopulmonary hospitalizations.

6.3.2.2 To compare the effect of high-dose influenza vaccine versus standard-dose vaccine on time to first occurrence of cardiovascular death or cardiovascular hospitalization in the ACHD subgroup of the INVESTED trial

6.3.2.3 To test the hypothesis that compared to non-ACHD patients enrolled in INVESTED trial, ACHD patients will have a lower rate of appropriate influenza vaccination in the season(s) preceding their enrollment (Appendix B).

6.4 Exploratory Objectives

6.4.1 To compare the effect of high-dose influenza vaccine versus standard-dose vaccine on time to first occurrence of all-cause death or cardiopulmonary hospitalization according to

effectiveness of vaccine relative to virulence of influenza strain and the quality of the match between influenza strain and vaccine within individual seasons

6.4.2 To compare the effect of high-dose influenza vaccine versus standard-dose vaccine on time to first occurrence of cardiovascular death or heart failure hospitalization

6.4.3 To compare the effect of high-dose influenza vaccine versus standard-dose vaccine on time to first occurrence of cardiovascular death, non-fatal MI, or non-fatal stroke

6.4.4 To compare the effect of high-dose influenza vaccine versus standard-dose vaccine on time to first occurrence of all-cause death or pulmonary hospitalizations

7.0 STUDY POPULATION

Eligibility waivers are not permitted. Subjects must meet all of the inclusion and no exclusion criteria to be enrolled in the study. Study vaccination may not be given until informed consent has been obtained, including presentation, discussion and signing of the informed consent form. .

**Inclusion Criteria**
1. Willing to give written informed consent and able to adhere to dosing and study visit and follow-up schedules
2. At least 18 years of age
3. Documented history of **<u>at least one</u>** of the below CV events:<br>a. Hospitalization for spontaneous MI (type 1 or type 2 event) (**<u>within one year of baseline visit</u>**)<br>b. Hospitalization for heart failure (**<u>within two years of baseline visit</u>**) but not currently acutely decompensated.
4. Fulfills at least **<u>one</u>** of the following additional risk factors:<br>a. Prior MI hospitalization (for subjects qualifying on HF hospitalization) or a second MI hospitalization for those qualifying based on MI)<br>b. Prior HF hospitalization (for subjects qualifying based on MI hospitalization) or a second HF hospitalization for those qualifying based on HF)<br>c. Age ≥ 65 years<br>d. Current or historical LVEF < 40%<br>e. Documented diagnosis (via ICD-9 code) of type I or type II diabetes mellitus (laboratory findings, e.g., elevated A1C, FPG, plasma glucose in the absence of a clinical diagnosis is not sufficient)<br>f. Current BMI ≥ 30<br>g. Documented history of renal impairment (eGFR ≤ 60 for at least 2 readings in the past year)<br>h. Documented history of ischemic stroke<br>i. Documented history of peripheral artery disease<br>j. Current tobacco smoker (smokes 1 or more cigarettes daily)
**Exclusion Criteria**
1. Known allergy, hypersensitivity (anaphylaxis), or Guillain-Barré Syndrome within 6 weeks after influenza vaccine, or severe allergy to egg protein
2. Any non-cardiac condition that in the opinion of the investigator would lead to life expectancy less than 9 months
3. Receipt of influenza vaccine during current influenza season
4. Any acute infection requiring antibiotics within 14 days of influenza vaccination (prophylactic antibiotics prior to dental or other procedures, or scheduled use of antibiotics for other types of prophylaxis does not exclude the subject). If an acute course of antibiotics is required, the patient may still participate in INVESTED 14 days after completing antibiotics.

5.	Known fever over 100 degrees Fahrenheit or 38 degrees Celsius within 7 days prior to influenza vaccination visit.
6.	Women who are pregnant or breast-feeding*
7.	Not suitable for study participation due to other reasons at the discretion of the investigator

* A woman who has not yet gone through menopause will be counseled to use adequate birth control measures (i.e., oral, implanted or barrier methods) while enrolled in this study. Pregnancy status will be assessed by self-report.

7.1 Accrual Goal

A total of 9,300 subjects will be enrolled from approximately 180 sites across the United States and Canada.

The study will enroll during 4 influenza seasons: one Vanguard season (N=500 subjects), and three additional seasons (N=2,933 each). INVESTED will utilize several “networks” of performance sites with established track records in clinical trials, which take advantage of existing infrastructures to improve efficiency with subject enrollment and follow up.

7.2 Recruitment Plan

Several recruitment strategies will be employed, and sites within each network will use a combination of methods depending on their capabilities. Specific recruitment strategies are as follows (non-comprehensive list):

1. Networks and sites with electronic health record abilities will query electronic health records based on study enrollment criteria and create screening lists for individual site PIs, which will be forwarded to site research personnel in the early summer months prior to each enrolling season.
2. Subjects will be enrolled prior to discharge from a hospitalization for acute heart failure or myocardial infarction if found no longer to be acutely decompensated.
3. Enrollment will occur as part of an outpatient visit in a cardiology or primary care clinic, cardiac rehab visit, or other clinical setting prior to routine influenza vaccination campaigns. The enrolling sites will generally be cardiology specialty clinics with a large volume of myocardial infarction or heart failure patients. Potential participants will be approached by a member of the health care team to assess interest in participating in INVESTED.
4. When feasible, letters will be mailed to potential subjects (identified either through electronic queries or through clinic patient lists), reminding them to get vaccinated during the fall months and informing them about the INVESTED study.
5. Potential subjects may undergo an initial eligibility screen by phone.
6. Individual sites may use additional strategies for which IRB approval will be obtained prior to implementation.

It is anticipated that a large portion of subjects will be identified prior to the start of influenza season, while the remainder will be enrolled from mid-August through January. Influenza vaccine is typically available at the end of August, and it is crucial to recruit individuals early prior to their routine receipt of influenza vaccine. Initial study visits will be scheduled in advance to commence when annual formulations of each study vaccine will be available for use in the trial.

8.0 INVESTIGATIONAL PRODUCT

8.1 Fluzone® Quadrivalent Influenza Vaccine (15 µg/strain, QIV)

Fluzone Quadrivalent influenza vaccine is licensed in the United States and Canada, and is indicated for active immunization of persons 6 months of age and older against influenza disease

caused by influenza virus subtypes A and type B. A single injectable sterile suspension 0.5 mL dose contains 15 μg of four viral strains for a total of 60 μg in one dose.

8.2 Fluzone® High-Dose Trivalent Influenza Vaccine (60 μg/strain, TIV)

Fluzone High-Dose influenza vaccine is licensed in the United States and Canada and is indicated for active immunization of persons 65 years of age and older against influenza disease caused by influenza virus subtypes A and type B. A single injectable sterile suspension 0.5 mL dose contains 60 μg of three viral strains for a total of 180 μg in one dose.

8.3 Properties

Both inactivated influenza vaccines are prepared from influenza viruses propagated in embryonated chicken eggs. The virus-containing allantoic fluid is harvested and inactivated with formaldehyde. Influenza virus is concentrated and purified in a linear sucrose density gradient solution using a continuous flow centrifuge. The virus is then chemically disrupted using a non-ionic surfactant, octylphenol ethoxylate (Triton® X-100), producing a “split virus”. The split virus is further purified and then suspended in sodium phosphate-buffered isotonic sodium chloride solution. The process uses additional concentration factor after the ultrafiltration step to obtain a higher hemagglutinin (HA) antigen concentration. Antigens from the three/four strains included in the vaccine are produced separately and then combined. The suspension for injection is clear and slightly opalescent in color.

8.4 Acquisition & Shipping

Sanofi Pasteur will provide both doses of Fluzone as 0.5 mL single-dose, pre-filled syringes. Biologics Inc. will be responsible for blinding the syringes and distributing study medications to the clinical sites. Each vaccine shipment will include a temperature-monitoring device to verify maintenance of the cold chain during transit, as well as a packing slip. On delivery of the product to the site, the person in charge of product receipt will follow the instructions given in the Manual of Procedures, including checking that the cold chain was maintained during shipment (i.e., verification of the temperature recorders). The contents of the shipment will then be reviewed and verified against the packing slip, and will be documented as instructed at the initiation visit. The temperature monitoring device will be read by the responsible person.

If the temperature-monitoring device indicates that the cold chain has been broken, the entire shipment must be immediately quarantined in refrigerated conditions (2–8°C). See the Manual of Procedures for further information on processing temperature deviations during shipment.

Sites will be responsible for ensuring appropriate receipt, use, disposition and reconciliation of the investigational product as outlined in the Manual of Procedures.

8.5 Product Storage and Stability

Investigational product will be stored under controlled temperature (between 2-8 °C) with excursions permitted to room temperature for vaccine administration. All investigational product must be stored in accordance with instructions outlined in the package insert. Investigational product must be stored separately from normal hospital stocks and must be stored in a securely locked area accessible only to authorized trial personnel until dispensed. The vaccines must not be frozen. The temperature must be monitored and documented on the appropriate form for the entire time that the vaccine is at the trial site. In case of accidental freezing or disruption of the cold chain, vaccines must not be administered, and the Investigator or responsible person should contact Biologics, Inc. for further instructions.

8.6 Accountability Procedures

Vaccine will be shipped to each site at the start of the enrolling season and at additional times throughout the influenza season if needed, dependent on that site’s recruitment. The person in

charge of product management at the site will maintain records of product delivery to the trial site, product inventory at the site, the dose given to each subject, and the disposal of or return of unused doses to Biologics, Inc. The DCC will verify each trial site's product accountability records against the record of administered doses in the eCRFs, the source documents, and the communication from the online randomization program.

In case of any expected or potential shortage of product during the trial, the Investigator or an authorized designee should alert Biologics, Inc as soon as possible, so that a shipment of extra doses can be arranged. In the event of a quality issue with a dose of vaccine, the site should quarantine the dose and contact Biologics for further instructions.

8.7 Dosage and Administration

Both Fluzone vaccines will be supplied as 0.5 mL single-dose, pre-filled syringes. No preparation of the vaccine is required. Both vaccines should be administered as follows:

- Visually inspect the syringe for particulate matter and/or discoloration prior to administration. If either of these conditions exist, the vaccine should not be administered.
- Identify the deltoid muscle (upper arm) and cleanse the injection site with alcohol (Figure 2)
- Insert needle (22 to 25 -gauge 1-inch needle recommended; 1 ½ inch needle recommended for those with BMI ≥ 30) at a 90 degree angle to the skin and inject entire contents of the syringe intramuscularly. Do not inject the vaccine subcutaneously or intravenously. Care should be taken to avoid administering the injection into or near blood vessels and nerves.
- Monitor the participant for at least 20 minutes post vaccination to ensure their safety. Appropriate medical equipment and emergency medications, including epinephrine (1:1000), must be available on site in the event of an anaphylactic or other immediate allergic reaction.

Figure 2: Intramuscular (IM) Injection Site for Adults

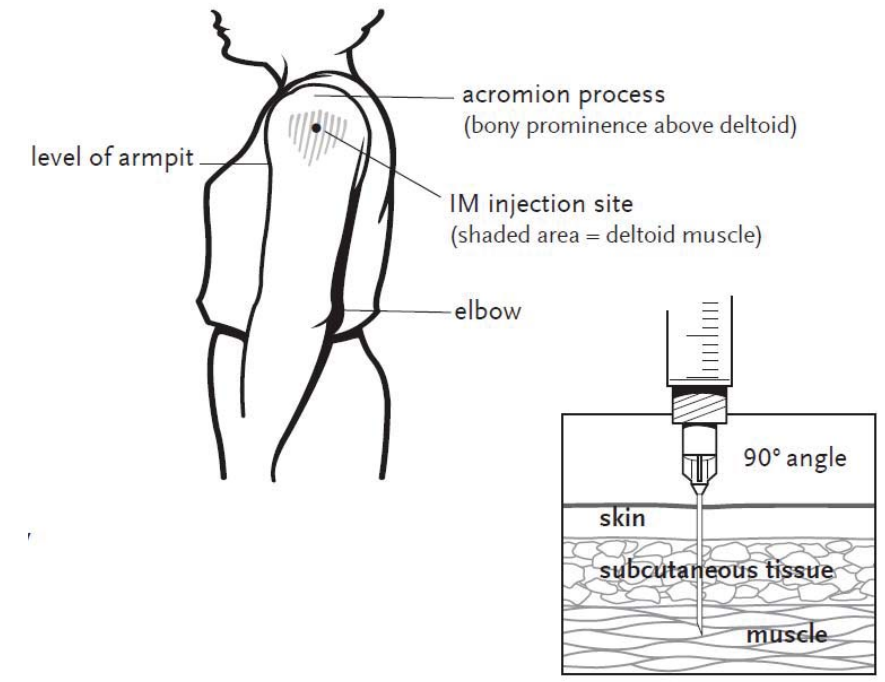

Administer injection in the central and thickest portion of the deltoid mucle – above the level of the armpit and approximately 2 inches below the acromion process. Do not inject too high (near the acromion process) or too low.

8.8 Adverse Reactions

Mild soreness at the injection site, headache, malaise, and muscle aches are the most commonly reported symptoms following influenza vaccination. As with all vaccines, recipients of the influenza vaccine incur a slight risk of experiencing a severe allergic reaction. Signs of serious allergic reaction can include breathing problems, hoarseness or wheezing, hives, paleness, weakness, a fast heartbeat, or dizziness. If reactions like this occur, it is typically within a few minutes to a few hours after the vaccine. There is some evidence of a link between some influenza vaccines and Guillain-Barré syndrome (GBS). GBS is a disease that can cause serious and even permanent damage to the nervous system. About 1 in 100,000 people per year develop GBS, a small number of these cases may be triggered by influenza vaccines.

8.9 Duration of Therapy

A single 0.5 mL influenza vaccine dose annually for up to three influenza seasons.

8.10 Duration of Follow-Up

Subject follow-up will occur up to four time points after each vaccine: 1 week post vaccination, 2-4 weeks post vaccination (only in a subset of subjects participating in the correlative study), during influenza season and in the summer. Each summer, subjects will be invited to return to participate in the next year of the study and each returning subject will be assigned to his/her initial vaccine strategy. If a subject does not wish to return for a study vaccination in subsequent influenza season, they will be taken off study at the date of last successful follow up contact during the year following randomization or registration and no longer followed.

8.11 Medications/Treatments Permitted and Not Permitted during the Trial

8.11.1 Rescue medication, emergency procedures, and additional treatment(s)

Epinephrine (1:1000) may be administered in the event of an anaphylactic or other immediate allergic reaction.

.

8.11.2 Allowed

Subjects will be treated with other medications at the discretion of their primary care provider. At study visits, current medications will be recorded. Subjects will be advised that they do not need to receive an additional (routine) dose of influenza vaccine in the current influenza season at any time during the study if undergoing annual study-drug vaccination. However, any subjects that do so will remain in the study and part of the primary analysis.

There are no dietary or lifestyle restrictions.

8.11.3 Participation in other research investigations

Concomitant participation in other research studies (including trials of investigational medications or devices) is allowed. If the subject is already participating in another trial prior to being enrolled in INVESTED, the site principal investigator should ensure that co-enrollment is allowed by the other study. Similarly, if an INVESTED subject wishes to participate in another study after being enrolled in INVESTED, this is allowable if allowed by the other study as determined by the site investigator.

8.12 Removal of Subjects from Study

All enrolled subjects should be followed according to the protocol specified visits and follow-up procedures. Subjects who did not receive study drug or are found to be ineligible after randomization or registration should be followed for the remainder of the year following randomization or registration. Case report forms should be submitted in a timely manner. Subjects may discontinue participation in the study at any time at their own request or at the discretion of the investigator for safety, behavioral or administrative reasons. The reasons(s) for discontinuation will be documented and may include:

- Subject withdraws from the study. Subjects who receive influenza vaccine and do not wish to be followed for that season's spring and summer ascertainment phone calls will be permanently withdrawn from the study effective the date they withdrew consent. The reason for withdrawal of consent will be documented for all subjects withdrawn from the study.
- Subject is unable or unwilling to comply with protocol requirements
- Subject experiences an adverse reaction that makes continuation unsafe
- Subjects who decline to continue receiving influenza vaccine. If the subject does not wish to receive influenza vaccine per protocol for subsequent influenza seasons, they will be taken off study at the date of last successful follow up contact during the year following randomization or registration and no longer followed.
- If a subject cannot be contacted they will be considered lost to follow up and removal from the study as of the date of last successful follow up contact and will no longer be followed.

All randomized participants and all events as defined in the protocol will be accounted for in the primary analysis.

8.13 Duration of Therapy

Enrolled subjects will receive vaccine and annual revaccination for up to 3 influenza seasons. All participants will be followed according to section 9.4. If a subject does not wish to return for study vaccination in subsequent influenza seasons, they will be taken off study as of the date of last successful follow up contact and no longer followed.

9.0 STUDY PROCEDURES

9.1 Screening/Baseline Visit

- Obtain written informed consent and medical release of information form
- Review medical records for past medical history (including recent hospitalization and previous influenza vaccinations) and current medications (will be also be assessed annually if enrolled)
- Reconfirm eligibility (if baseline visit is on a later date than the screening visit)
- Collect medical history, social history, baseline questionnaires (i.e frailty survey; NYHA/CCS functional status), demographics and contact information for follow-up, including the patient's social security number (US only) or provincial health insurance number (for Canada only)- when permissible according to local ethics regulations, the names and contact information of two family members or friends that could be contacted if the subject is unreachable
    - For participants with documented evidence of adult congenital heart disease (ACHD) participating in the correlative ACHD substudy, additional medical and surgical history will be collected.
- Review medical records for height, weight, and temperature (if available)
- Patients must not be given study vaccination prior to Randomization
- Blood will be drawn for baseline measurement of antibody titers and other biomarkers in a subset of consenting subjects
- Randomize subject via the internet using the Statistical Registration and Randomization System
    - The StaRRS randomization software will return a treatment code corresponding to either high-dose or standard-dose vaccine
    - Labels containing the treatment allocation code will be on the drug packet. Verify correct assignment.
- Dispense and administer vaccine (all efforts should be made to vaccinate same day as randomization)
- Monitor subject for at least 20 minutes post vaccination for injection site and allergic reactions
- Dispense 7 day AE diary with instructions for reporting recorded information at the one week follow up phone call
- Provide a proof of vaccination letter to those who may require it
- Schedule one-week follow-up contact

9.1.2 PATIENTS RETURNING FOR YEAR 2 AND 3

- Review medical records for past medical history (including recent hospitalization and previous influenza vaccinations) and current medications
- Confirm that the patient does not meet exclusion criteria
- Collect medical history, social history, questionnaires (i.e frailty survey; NYHA/CCS functional status), demographics and confirm contact information for follow-up, the names and contact information of two family members or friends that could be contacted if the subject is unreachable

- For participants with documented evidence of adult congenital heart disease (ACHD) participating in the correlative ACHD substudy, additional medical and surgical history will be collected
- Review medical records for height, weight and temperature) (if available)
- Blood will be drawn for baseline measurement of antibody titers and other biomarkers in a subset of consenting subjects.
- *Register* subject via the internet using the Statistical registration and Randomization System **before** administering vaccination
  - The StaRRS software will return a treatment code corresponding to the vaccine dose to which the subject was previously randomized
  - Labels containing the treatment allocation code will be on the drug packet. Verify correct assignment
- Dispense and administer vaccine (all efforts should be made to vaccinate same day as registration)
- Monitor subject for at least 20 minutes post vaccination for injection site and allergic reactions
- Dispense 7 day AE diary with instuctions for reporting recorded information at the one week follow up phone call
- Provide a proof of vaccination letter to those who may require it
- Schedule one-week follow-up contact

9.2 Follow-up

One week following the randomization visit, subjects will be contacted by a member of the study team by phone to collect their 7 day diary information. If subjects are not reached one week following vaccination study team will continue attempts to obtain day 7 diary information. Thereafter, surveillance for hospitalization or death will include one telephone call during influenza season, and another phone call in the summer following influenza season. Subjects will be asked to report any hospitalizations since the last contact, including the name of the hospital, the reasons for the hospitalization, and the dates. . To the extent possible, collection of 7-day diary information and ascertainment of hospitalizations should be performed by a different study team member than the study team member who administered the study vaccination. In between scheduled contacts, subjects will be asked to inform local site personnel of hospitalizations. Subjects will be able to report hospitalizations by phone.

If the subject cannot be reached for their follow up calls (during influenza season or in the summer) at the scheduled time, the interviewer will make additional attempts at different times of day over the next few days and may also send a certified letter to the subject. If two consecutive telephone interviews are missed, the interviewer will contact one or both of the two contacts provided at the time of enrollment. The interviewer will continue to attempt to contact a subject until the end of the summer following vaccination unless the subject withdraws consent. Sites are supported in following their institutional policies

During the summer phone call, local coordinators will call subjects to ascertain for hospitalizations or death, and assess the subject’s willingness to receive influenza vaccine the following season. Subjects who are interested in receiving influenza vaccination will have their vaccination visit scheduled by local site personnel. Subjects who do not wish to continue receiving study vaccination in subsequent influenza seasons will be taken off study as of the date of last successful follow-up contact during the year following randomization or registration.

CONFIDENTIAL

To maximize accuracy of outcome ascertainment compared with details obtained through clinical follow up, subject medical records and administrative health insurance claims data will be periodically reviewed during, at the end, and after the trial period to document vital status, potential cause of death, and hospitalizations. Where permissible, the CCC and DCC will link trial data in accordance with privacy-protected and data-secure health insurance claims data agencies (in Canadian sites only) in order to minimize loss to follow-up and provide enhanced detection of safety and outcomes as recommended by guidance documents from Canadian regulatory health authorities.

For all subjects, when vital status cannot be ascertained study personnel may search public death records for confirmation and cause of death.

9.2.1 Year 2 and 3 Follow Up
Returning patients in years 2 and 3 following year 1 procedures after their vaccine visit.

9.3 Correlative Studies Procedures

9.3.1 Immune Response Sub-study, for a subset of subjects only, after the Vanguard year
This sub-study is optional for sites. Subjects participating in the immune response sub-study (up to 1,000 per year) will be scheduled for a follow-up visit between two-four weeks (+ 4 days) post-vaccination. Subjects unable to participate for a follow up blood draw will not be included in the immune response sub-study. Subjects may participate in the immune response sub-study multiple years.

Blood will be drawn to assess antibody titers. Among a subset of sub-study subjects who agree to it, additional blood will be collected for future research about other biomarkers, and genetics.

9.3.2 ACHD Sub-study
This sub-study is optional for sites. Enrolled participants (N=500) will follow study procedures as specified in the INVESTED protocol. Total duration of subject participation will be up to 3 influenza seasons. No additional visits or procedures are required for this sub study. The ACHD case report form should be completed for each enrolled patient within 1 week following study registration. Patients will be registered using Frontier Science's web-based Statistical Registration and Randomization system. Registration can be completed as the patient is initially randomized to the INVESTED Trial, or Concurrent with registration for INVESTED returning years 2 or 3. Participants previously randomized to and participating in the INVESTED trial may be retroactively entered on this sub study concurrent with registration for returning years 2 and 3 (Appendix B).

9.4 Schedule of Time and Events Table

Measurement	Screening[1] Visit	Baseline Visit (August - January)	Week 1 Phone Call (+ 4 days)	Week 2-4 Visit (+ 4 days)[3]	During Influenza Season Phone Call	Summer Phone Call	Yrs 2 & 3 Baseline[4] (August - January)
Informed Consent	X						
Demographics & History[2]	X	X					X
Inclusion/Exclusion	X	X					X

CONFIDENTIAL

Current Medications	X	X					X
Blood Draw[3]		X[5]		X[5]			X[6]
Vaccine Administration		X					X
Assessment of vaccine-related reactions		X	X				X
Cardiopulmonary Event Assessment				X	X	X	
Year 2&3 Visit Scheduling						X	

1. Screening and baseline procedures may be completed at one visit, followed by randomization and vaccine administration
2. History includes previous vaccinations
3. Immune endpoints at Baseline and at Week 2-4 visit (e.g., geometric mean titers post-vaccination, change in antibody titers between 2-4 weeks post-vaccination, seroconversion, seroprotection, and B-type vaccine antigens 2-4 weeks post-vaccination) will be assessed in subset of up to 3,000 subjects at participating sites.
4. Years 2 and 3 follow year one procedures after baseline visit
5. Optional additional blood draw at either Baseline or Week 2-4 visit for future research about biomarkers and genetic markers.
6. Returning subjects who elect to participate in the immune response sub-study in year 2 and 3 have sub-study blood draw

10.0 OUTCOME MEASURES

10.1 Primary Efficacy Endpoint

10.1.1 Time to first occurrence of death or cardiopulmonary hospitalization within each enrolling season

10.2 Secondary Efficacy Endpoints

10.2.1 Total (first and recurrent) cardiopulmonary hospitalizations or death

10.2.2 Time to first occurrence of cardiovascular death or cardiovascular hospitalization within each enrolling season

10.2.3 Time to first occurrence of death or cardiopulmonary hospitalization across all enrolling seasons

10.2.4 Time to first occurrence of the individual components of the primary endpoint

10.3 Exploratory Efficacy Endpoints

10.3.1 Time to first occurrence of all-cause death or cardiopulmonary hospitalization according to effectiveness of vaccine relative to virulence of influenza strain and the quality of the match between influenza strain and vaccine within individual seasons

10.3.2 Time to first occurrence of cardiovascular death or heart failure hospitalization

10.3.3 Time to first occurrence of cardiovascular death, non-fatal MI, or non-fatal stroke

10.3.4 Time to first occurrence of all-cause death or cardiopulmonary hospitalizations

10.4 Primary Safety Endpoint

10.4.1 Incidence of vaccine injection site reactions (described in section 11.0)

10.5 Secondary Safety Endpoints

10.5.1 Incidence of vaccine-related adverse events and serious adverse events

10.6 Correlative study endpoints

CONFIDENTIAL

10.6.1 Immune Response Sub-Study (up to 3,000 subjects)

10.6.1.1 Geometric mean titers post vaccination

10.6.1.2 Change in antibody titers at 4 weeks post-vaccination from baseline

10.6.1.3 Seroconversion, i.e. 4-fold rise in antibody concentrations from baseline to A/H1N1, A/H3N2, and B-type vaccine antigens

10.6.1.4 Seroprotection, i.e. antibody titer levels $\geq$ 1:40 to the A/H1N1, A/H3N2, and B-type vaccine antigens 4 weeks following influenza vaccination. Seroconversion and seroprotection to A/H1N1, A/H1N3 strains and the B-Yamagata lineage.

10.6.2 ACHD Sub-Study

10.6.2.1 Time to first occurrence of death or cardiopulmonary hospitalization within each influenza season in the ACHD population. (Appendix B)

11.0 ASSESSMENT OF SAFETY

11.1 Specification of Safety Parameters

11.1.1 Definition of Adverse Events (AE)

An AE is any untoward or unfavorable medical occurrence in a human subject, including any abnormal sign (for example, abnormal physical exam or laboratory finding), symptom, or disease, temporally associated with the subject's participation in the research, whether or not considered related to the subject's participation in the research.

11.1.2 Definition of Serious Adverse Events (SAE)

A SAE is one that meets one or more of the following criteria:

- Results in death
- Is life-threatening (places the subject at immediate risk of death from the event as it occurred)
- Results in inpatient hospitalization or prolongation of existing hospitalization
- Results in a persistent or significant disability or incapacity
- Results in a congenital anomaly or birth defect
- An important medical event that may not result in death, be life threatening, or require hospitalization may be considered an SAE when, based upon appropriate medical judgment, the event may jeopardize the subject and may require medical or surgical intervention to prevent one of the outcomes listed in this definition.

As death and hospitalizations for cardiovascular and pulmonary reasons are considered clinical endpoints for this trial, they will be reported in clinical study reports and publications as clinical endpoints.

11.1.3 Definition of Unanticipated Problems (UP)

An AE is considered unanticipated, when either the type of event or the severity of the event is not listed in the protocol or package insert/product monograph.

11.2 Classification of an Adverse Event

11.2.1 Severity of Event

- Mild: no intervention required; no impact on activities of daily living (ADL)
- Moderate: minimal, local, or non-invasive intervention indicated; moderate impact on ADL
- Severe: significant symptoms requiring invasive intervention; subject seeks medical attention, needs major assistance with ADL

CONFIDENTIAL

11.2.2 Relationship to Study Agent

- Definitely Related – There is clear evidence to suggest a causal relationship, and other possible contributing factors can be ruled out. The clinical event, including an abnormal laboratory test result, occurs in a plausible time relationship to drug administration and cannot be explained by concurrent disease or other drugs or chemicals.

- Probably Related – There is evidence to suggest a causal relationship, and the influence of other factors is unlikely. The clinical event, including an abnormal laboratory test result, occurs within a reasonable time after administration of the drug, is unlikely to be attributed to concurrent disease or other drugs or chemicals.

- Possibly Related – There is some evidence to suggest a causal relationship (e.g., the event occurred within a reasonable time after administration of the trial medication). However, other factors may have contributed to the event (e.g., the subject's clinical condition, other concomitant events). Although an AE may rate only as "possibly related" soon after discovery, it can be flagged as requiring more information and later be upgraded to "probably related" or "definitely related," as appropriate.

- Unlikely to be related – A clinical event, including an abnormal laboratory test result, whose temporal relationship to drug administration makes a causal relationship improbable (e.g., the event did not occur within a reasonable time after administration of the trial medication) and in which other drugs or chemicals or underlying disease provides plausible explanations (e.g., the subject's clinical condition, other concomitant treatments).

- Not Related – The AE is completely independent of study drug administration, and/or evidence exists that the event is definitely related to another etiology. There must be an alternative, definitive etiology documented by the clinician.

11.2.3 Follow-up of Subjects after a Reportable SAE

SAEs with a causal relationship to trial participation must be followed until the event or its sequelae resolve or stabilize to a level acceptable to the local investigator.

11.2.4 Expectedness

Expected events are those that have been previously identified as resulting from administration of the investigational product. An AE is considered unexpected, when either the type of event or the severity of the event is not listed in the protocol or package insert.

11.3 Time Period and Frequency for Event Assessment and Follow-Up

All adverse events temporally related (within 1 week) to vaccine administration will be collected on appropriate CRFs. Specific adverse events of interest (see section 11.4.4) will be queried.

All serious adverse events that are possible trial endpoints, including death and all hospitalizations, will be collected on appropriate endpoint collection forms. All other serious adverse events not covered by endpoint collection forms will be collected on appropriate SAE CRFs. CRFs will be completed at the time the event becomes known to study investigators, either by notification by the subject, or during influenza season surveillance, at which time

CONFIDENTIAL

investigators will inquire about the occurrence of events since the last time of contact. Serious adverse events and trial endpoints (death or hospitalization) will be collected until July 31 of the influenza season.

All suspected unexpected serious adverse reactions (SUSARs) will be collected on appropriate CRFs during the course of the trial. All SUSARs will be collected at the time they are known to study investigators and may be subject to specific reporting requirements by health authorities or local IRBs.

Non-serious adverse events not temporally related to study vaccine administration (> 1 week) and not suspected to be related to study vaccine administration will be collected locally only, per GCP guidelines.

11.4 Reporting Procedures

11.4.1 Adverse Event Reporting
All adverse events that occur within 1 week of study vaccine administration will be collected and specific adverse events of interest (see section 11.4.4) will be queried. Non-serious adverse events not temporally related to study vaccine administration (> 1 week) and not suspected to be related to study vaccine administration will not be collected.

11.4.2 Serious Adverse Event Reporting
All serious adverse events that are possible trial endpoints, including death and all hospitalizations, will be collected on appropriate endpoint collection forms. All other serious adverse events not covered by endpoint collection forms will be collected on appropriate SAE CRFs. CRFs will be completed at the time the event becomes known to study investigators, either by notification by the subject, or during influenza season surveillance, at which time investigators will inquire about the occurrence of events since the last time of contact. Serious adverse events and trial endpoints (death or hospitalization) will be collected until July 31 of the influenza season.
This trial has received an IND exemption, and serious adverse events will not be required to be reported to FDA. Serious, unexpected adverse drug reactions will be reported to Health Canada in an expedited manner as required (section 11.4.3). SAEs will be reported to local IRBs per local IRB requirements.

11.4.3 Unanticipated Problem Reporting
All suspected unexpected serious adverse reactions (SUSARs) will be collected on appropriate CRFs during the course of the trial. All SUSARs will also be collected at the time they are known to study investigators. There is no requirement for SUSAR reporting to FDA because of the IND exemption, however, all fatal or life threatening SUSARs will be reported to Health Canada as soon as possible but no later than 7-15 calendar days after the event becomes known to study investigators. All non-fatal or non-life threatening SUSARs must be filed as soon as possible but no later than 15 calendar days after the event becomes known to study investigators. All SUSARs will be reported to Sanofi-Pasteur and to local IRBs as per local IRB requirements.

11.4.4 Adverse Events of Interest
The following events occurring within one week of study vaccine administration are considered events of special interest and will be specifically queried:

- Injection site reactions (pain, redness, or swelling at injection site)
- Myalgia

CONFIDENTIAL

- Fever
- Malaise
- Headache

The following important medical events will be considered SAEs and reported according to section 11.4.3:

- Guillain-Barré syndrome
- Bell's palsy
- Encephalitis/myelitis
- Optic neuritis
- Stevens-Johnson syndrome
- Toxic epidermal necrolysis

11.5 Study Termination Rules

The DSMB may recommend stopping the study early if the incidence of safety events in the high-dose arm is unacceptably higher than in the standard-dose arm or for clear efficacy results. For additional details, see Section 13.14 on Stopping Rules for Termination of the Trial.

11.6 Unblinding Procedure

Unblinding will be done in emergent circumstances where the identity of the study medication needs to be known. All efforts will be made to maintain blinding except in the case of urgent medical necessity. If a subject needs to be unblinded, the study staff should contact their assigned medical monitor within 24 hours. The study site must document who broke the blind and reason, and report the event to a member of the trial's executive leadership team (Dr. Solomon or Dr. Vardeny) within 24 hours. Contact the medical monitor first, who will instruct the data coordinating center to unblind.

Unblinded subjects will not be re-vaccinated in subsequent influenza seasons as part of the trial and taken off study as of the date of the summer phone call contact following randomization or registration.

11.7 Safety Oversight

Safety oversight will be under the direction of a DSMB composed of individuals with the appropriate expertise, including cardiovascular disease, pulmonary disease, infectious disease, statistics, and ethics. The DSMB will meet at least semiannually to assess safety and efficacy data on each arm of the study. The DSMB will operate under the rules of an approved charter that will be written and reviewed at the organizational meeting of the DSMB. At that time, each data element that the DSMB needs to assess will be clearly defined. The DSMB will provide its input to the NHLBI project office, and the study executive committee.

12.0 CORRELATIVE (IMMUNE RESPONSE, BIOMARKER AND DNA) STUDIES

Antibody titers to influenza vaccine antigens, seroprotection, and seroconversion will be assessed in a subgroup of subjects (up to 3,000 subjects, approximately 1,000 subjects from each of the three influenza seasons) to test the hypothesis that a higher influenza vaccine dose will result in more pronounced humoral immune response, evidenced by greater mean titers post-vaccination and higher antibody titer changes from baseline, and to test the hypothesis that higher antibody concentrations will be associated with the reduced rate of the composite of all-cause death and cardiopulmonary hospitalization. Participation in correlative studies is optional.

CONFIDENTIAL

Additional blood will be collected from a further subset of correlative study subjects for future research about biomarkers and DNA.

A subset of approximately 35 sites from geographically diverse regions in the US and Canada will be asked to participate in the humoral immune response sub-study.

12.1.1 Sample Collection Guidelines

Subjects electing to participate in the optional correlatives studies that become available after the Vanguard year will contribute two blood samples, one at baseline and a second sample two-four weeks after vaccination. At the first time point, 10 ml of blood will be collected in serum tubes for immune function analyses. At the second time point, 20 ml will be collected for immune function testing. At one of these timepoints, an additional 10 ml of blood will be collected in DNA collection tubes (only if the subject consents to banking of their blood for genetic testing).

Following the blood draw, the sampling tube should be stored at room temperature for a minimum of 60 minutes and a maximum of 2 hours to allow the blood to clot before centrifugation. The tube must be stored vertically and not shaken. Beyond 2 hours, the sampling tube must be refrigerated at a temperature of 2°C to 8°C and must be centrifuged within a maximum of 24 hours. After being allowed to clot for a minimum of 60 minutes to a maximum of 2 hours at room temperature (or after being refrigerated for 2 to 24 hours after collection), blood samples for serum antibody response assessment will be centrifuged before being divided into at least 3 aliquots, if possible. Sera will be placed in cryovials labeled with the study ID, collection number (#1 or #2), and collection season (i.e. 2016/17). Cryovials will be frozen at -20 degrees (or colder) and stored at the study sites. Once blood collection is completed or at the end of each influenza season, samples will be batch shipped to Brigham and Women’s Hospital (U.S. sites) and Peter Munk Cardiac Centre (Canadian sites) for further analysis.

12.1.2 Assay Methodology for immune response studies

Brigham and Women’s Hospital and Peter Munk Cardiac Centre will ship two cryovials from each subject, labeled as described above, to Sanofi Pasteur, Inc. in Pennsylvania for analysis, accompanied by no other information. A hemagglutination inhibition assay (HIA) will be used to measure influenza antibody concentrations following immunization. HIA will be performed in duplicate using standard microtiter techniques. Briefly, influenza virus–induced agglutination of guinea pig red blood cells will be inhibited by antibodies present in the human serum. Serial dilutions of the human sera will be made. Titrated influenza antigen will be incubated with the serum dilutions for 30 minutes. Guinea pig red blood cells (50 µL of 0.5% in phosphate buffered saline) will be added and incubated for 45 minutes. The dilution of serum that no longer inhibits hemagglutinin signifies the influenza antibody titer. Sanofi Pasteur, Inc. will destroy the samples once analysis is complete.

12.1.3 Specimen Banking

Once the study has ended, serum samples of subjects who did not agree to an additional blood collection for DNA analysis, banking, and future use will be anonymized by destroying the key linking Subject ID to subject identity. An aliquot will be sent to and banked at the NIH BioLINCC biorepository and will be managed per NIH Data sharing policies. This banking is not optional for subjects who agreed to participate in the

CONFIDENTIAL

correlative studies (substudy). The remaining samples from these subjects will be destroyed. Serum samples sent to NIH will not be used for DNA analysis.

The serum and whole blood of correlative study subjects who agreed to the additional blood collection for DNA, banking, and future use will be similarly anonymized. If funding becomes available in the future, one aliquot of each anonymized sample will be shipped commercially to NIH for banking and future unspecified research, including genetic testing and GWAS. NIH may place resulting genetic data in its own Database of Phenotypes and Genotypes or share with other federal genetic data repositories. The rest of the anonymized samples from these subjects will remain at Brigham and Women's Hospital (for specimens from US sites) and at the Peter Munk Cardiac Centre (for specimens from Canadian sites) for future unspecified research, including genetic testing (if funding becomes available) and creation of cell lines.

12.2 ACHD Sub-study

The ACHD sub-study will aim to recruit at least 500 patients of the total number of anticipated INVESTED subjects. Study procedures in the parent INVESTED protocol will be followed. No additional visits or procedures are required for this companion ACHD study. The ACHD case report form should be completed for each enrolled patient within 1 week following study registration. Patients previously randomized to and participating in the INVESTED trial may be retroactively entered on this sub study concurrent with registration for returning years 2 and 3. (Appendix B).

13.0 STATISTICAL CONSIDERATIONS

13.1 Statistical Analysis Plan

All statistical data analyses will be performed using SAS (SAS Institute, Inc., Cary NC) and R (R Foundation for Statistical Computing, Vienna, Austria. http://www.R-project.org/).

13.2 Analysis Population

Because the effects of vaccination last for at least a calendar year, there will be no typical adherence issues with vaccination at enrollment. However, we expect some subjects will not be revaccinated according to randomization in subsequent influenza seasons. We will therefore employ a modified intention-to-treat (mITT) analysis in which subjects' clocks will be reset 2 weeks after influenza vaccination, and primary endpoint events will be counted until July 31 of that year. Each subject can therefore contribute primary endpoint events in each season they are vaccinated. Although a subject's experiences from year to year may be correlated, for simplicity, each subject's contribution for each enrolling season will be treated as independent. We will assess the independence of the primary endpoints from year to year in a sensitivity analysis as described in Section 13.4. The usual intent-to-treat (ITT) principle for the analysis of randomized controlled trials will be followed for the assessment of balance of baseline subject characteristics and reporting of adverse events and for recurrent events analyses described below in Sections 13.4 and 13.5.

13.3 Analysis of Baseline Characteristics

We will assess the balance in the randomization groups with regard to subject characteristics including cardiovascular disease measures. The subjects will be compared on each characteristic between the randomized vaccine arms using methodology appropriate to the measure. More specifically, baseline subject characteristics will be summarized using descriptive statistics such as mean and standard deviation or median and interquartile range (IQR) for quantitative measurements and frequency and proportion for binary or categorical measurement and using graphics such as box plots and empirical distribution functions and assessed for balance between the two dose groups.

13.4 Analysis of Primary Endpoints

The primary efficacy analysis will be performed according to the mITT principle described above on the primary endpoint of the time to first occurrence of all-cause death or cardiopulmonary hospitalization within each enrolling season (See 6.1). The event accrual period will begin 2 weeks following receipt of influenza vaccine and continue until the summer phone call (July 31st) follow-up using standard survival analysis methods. The primary efficacy analysis will be based on a two-sided log-rank test at a significance level of 0.05, stratified by influenza season. The Kaplan-Meier method will be used to estimate the survival distribution for the time to first event for the composite of all-cause death or cardiopulmonary hospitalization.[22] An unadjusted estimate of the hazard ratio and confidence interval will be obtained using a Cox proportional hazards model with only treatment as a model term, stratified by influenza season.[23] Because we will utilize a randomization-once strategy for subjects who remain in the trial for multiple seasons, this approach will allow us to test the hypothesis that a strategy of high-dose influenza vaccine over multiple seasons will be superior to standard-dose vaccine, without running the risk that re-randomization would dilute a potentially cumulative effect. To examine this hypothesis, a secondary analysis will be a standard ITT analysis of the time from randomization to first occurrence of all-cause death or cardiopulmonary hospitalization across all enrolling seasons (See 6.2.3). In order to account for potential differential survivorship bias and bias due to differential drop-out after the initial randomization, we will use principal stratification, matching based on propensity score or inverse probability of treatment weighting for adjusted Kaplan-Meier estimator and log-rank test as a sensitivity analysis.[24-26] Another secondary efficacy analysis will be based on a Cox proportional hazards model with age group (< 65 or ≥ 65 years old), baseline cardiovascular (CV) risk group (AMI or HF), and treatment (high-dose or standard-dose influenza vaccination) as model terms, stratified by influenza season, to obtain an adjusted hazard ratio with confidence intervals, while adjusting for the following covariates: past vaccination history/pattern to adjust for the theoretical possibility of interference between successive vaccinations, both prior to randomization and after randomization, and match between vaccine and circulating influenza strains, and the interaction between treatment and match for circulating B (Victoria)-lineage that is included only in the standard-dose QIV (binary), based on influenza typing and subtyping data from Canada and the US to account for the differences in B vaccine antigens present only in the standard-dose QIV. A secondary "in season" analysis will also be undertaken, limited to an evaluation of efficacy during influenza season with start and end of season defined according to the Centers for Disease Control and Prevention (CDC) and Public Health Agency of Canada surveillance system. For example, we will use information provided in the CDC's Flu View Report which is updated on a weekly basis http://www.cdc.gov/flu/weekly/. For each state, we will use the point at which influenza transitions from "sporadic" to "local" on the graphic "Geographic Spread of Influenza as Assessed by State and Territorial Epidemiologists" or by using the point of transition from "minimal" to "low" activity on the "ILINet State Activity Indicator Map". A similar approach will be utilized for each province in Canada.

In order to assess the independence of the primary endpoints from year to year in individuals receiving influenza vaccines more than once, the frailty model version of the Cox proportional hazards regression will be evaluated.[27] In case the independence assumption is not tenable, we will estimate intra-subject correlation from year to year using the method of Prentice and Cai.[28]

13.5 Analysis of Secondary Endpoints

Secondary endpoints will include total (first and recurrent) cardiopulmonary hospitalizations or all cause-death during the subject's entire study participation duration, the composite of cardiovascular death or cardiovascular hospitalization within each enrolling season, the composite of all-cause death or cardiopulmonary hospitalization across all enrolling seasons, and individual components of the primary endpoint, including time to all-cause death and time to

CONFIDENTIAL

first occurrence of cardiopulmonary hospitalization. Time to composite endpoints and times to individual components of the composite endpoints will be analyzed similarly as the primary endpoint in Section 13.4 with individual components of the composite endpoints that are non-terminating events analyzed using methods for competing risks.[29] Recurrent events analysis will be performed for recurrent non-terminating events across all enrolling seasons.[30-32] For all analyses, two-sided p-values < 0.05 will be considered statistically significant. In addition, the rate of cardiopulmonary hospitalization with death as competing risk will be analyzed using nonparametric and semi-parametric analyses based on the mean frequency function defined as the marginal mean of the cumulative number of cardiopulmonary hospitalizations over time subject to a terminal event of death.[33,34]

13.6 Analysis of Safety Endpoints.

The number and percentage of subjects experiencing influenza vaccine related adverse events (e.g. injection site reactions, systemic side effects including headache, myalgia, and fever) and SAEs will be summarized by system organ class and by treatment arm. The safety endpoints will be summarized using frequency and proportion and compared between the two dose groups using nonparametric methods such as chi-square or Jonckheere-Terpstra tests.[35]

13.7 Analysis of Immune Outcomes (Correlative Study Endpoints)

Antibody titers and other immune responses such as the incidence of seroconversion and seroprotection in A/H1N1, A/H3N2 and B-type vaccine antigens will be summarized with descriptive statistics such as mean and standard deviation or median and IQR for quantitative measures and frequency and proportion for qualitative measures for baseline and week 4 post-vaccination along with graphics such as bar plots and compared between the two treatment arms using two-sample tests. Clinical outcomes will be compared between those with and those without seroconversion and seroprotection in A/H1N1, A/H3N2 and B-type vaccine antigens using log-rank tests. Cox proportional hazards regression analysis will be performed for the primary endpoint of the composite of all-cause death or cardiopulmonary hospitalization with the geometric mean titer across vaccine strains post-vaccination as a model term, instead of individual antibody titers, to avoid issues of collinearity, stratified by influenza season, while adjusting for other covariates including treatment and other immune responses and the interaction between treatment and match for circulating B (Victoria)-lineage that is included only in the standard-dose QIV (binary), based on influenza typing and subtyping data. The hazard ratio for each doubling of the geometric mean titer will be estimated along with confidence intervals from Cox proportional hazards regression models. Given that the traditional definition of seroprotection, namely HAI titers ≥ 40, may be an inadequate estimate of a protective threshold during years of poor match between vaccine antigens and circulating strains,[36] we will also assess seroprotection defined as HAI ≥ 80, 160, and 320 as exploratory analyses.

13.8 Additional Analyses

In addition to the analysis of the clinical and immune outcomes, we will evaluate the association between antibody titers post-vaccination and subsequent hospitalizations using Poisson or negative binomial regression models to investigate the association of number of days in hospital per month on the log of the titers, controlling for treatment and other important covariates.

13.9 Subgroup Analysis

Many baseline characteristics are known to be prognostic or suspected to be confounders for the clinical outcomes in the study population with high-risk cardiovascular disease. They include age, sex, race/ethnicity, smoking status, obesity (BMI≥30), baseline CV risk group (AMI vs HF), diabetes, renal dysfunction, and use of statin and beta-blocker medications. Influenza season is also known to affect the clinical outcome. Internal consistency of the primary analysis will be assessed in subgroups defined by these and other baseline characteristics. Heterogeneity of

CONFIDENTIAL

efficacy will be assessed using interaction tests of treatment by each of these baseline covariates and by influenza season. Any interaction test resulting in a heterogeneity p-value < 0.10 will be further evaluated for clinical plausibility of effect modification.

13.10 Missing data

This trial has many safeguards in place for assuring nearly complete data; therefore, the extent of missing data is expected to be small. Nevertheless, missing data may be associated with the outcome. For example, subjects with fewer hospitalizations or symptoms may travel during influenza season and be unavailable for follow-up calls. Most methods for handling missing data, such as multiple imputation, assume that the data are missing at random which would not be valid in this study. Where data are missing, sensitivity analyses will be performed using several assumptions to evaluate the sensitivity of the statistical results to the possible effects on the non-completers. To the extent possible, the reasons for missing data will be documented and evaluated. Assumptions about the missing data mechanism will be assessed using this information; these assumptions will be used to impute missing values under a variety of scenarios. In multiple imputations, the missing values are replaced with values consistent with several possible scenarios. If the missing data are extensive, model-based approaches will be considered to estimate effects under various assumptions regarding missingness.[37] Missing data analysis will follow the guideline promulgated in the National Research Council report.[38]

13.11 Interim Analysis

Formal interim analyses for efficacy will be performed twice: at the end of each enrolling season before the final analysis (which will occur at the end of the third enrolling season of 2019-2020) using the Lan-DeMets type I error spending function approach according to the O'Brien-Fleming group sequential method.[39,40] The following table shows the O'Brien-Fleming group sequential boundary based on the design assumptions:

End of Influenza Season Analysis	Information Fraction	Number of Primary Endpoint Events	Upper Efficacy Boundary	Nominal p-value
2017-2018	0.259	336	4.25	<0.0001
2018-2019	0.599	776	2.67	0.0075
2019-2020	1.000	1,296	1.98	0.0476

The Lan-DeMets approach allows flexibility needed in interim analysis of time to event data by using information time rather than calendar time to calculate the amount of type I error probability to spend at each interim analysis. Information time is defined as the number of accumulated events at interim analysis divided by the 1,296 events expected in the trial (See below in Section 13.12).

13.12 Number of Subjects to be Enrolled

The enrollment target is 4,650 subjects per treatment arm, for a total of 9,300 subjects. The assumed treatment effect size of high-dose vs. standard-dose influenza vaccine is derived from our meta-analysis of randomized trials of relatively healthy outpatients comparing these two active vaccination treatments, with an estimated risk reduction of 27% for the composite endpoint. After conservatively diluting the treatment effect among those with active heart disease by 35%, treatment with high-dose influenza vaccine is expected to result in an 18% risk reduction, i.e., a hazard ratio of 0.82, in all-cause death or cardiovascular hospitalizations, with an anticipated similar magnitude for all-cause death and cardiopulmonary hospitalizations. Based on data from contemporary clinical trials of patients with coronary heart disease or heart failure (see Appendix A),[41-49] the event rate for the primary endpoint is estimated to be 9% during the subject's 1st enrolling season following randomization for each subject, reducing to

CONFIDENTIAL

8% during each subject's 2nd enrolling season, and 7% during each subject's 3rd enrolling season after vaccination, with 30% of the primary composite endpoint being death and 70% being cardiopulmonary hospitalization. Considering a follow-up to the end of summer phone call on July 31 (before the next influenza season) and a conservative 30% rate of not being vaccinated in each subsequent influenza seasons, a trial of 9,300 subjects with 500 subjects for the Vanguard year in 2016-2017 and 2,933 new subjects in each of the three influenza seasons in 2017-2018, 2018-2019 and 2019-2020 is projected to result in 45, 291, 440 and 519 primary endpoint events by the end of the 2016-2020 enrolling seasons for a total of 1,296 events. Assuming two interim analyses for efficacy using the O'Brien-Fleming group sequential method at the end of 2017-2018 and 2018-2019 influenza seasons, the trial will have power of 0.943 to detect an 18% risk reduction or power of 0.913 to detect a 17% risk reduction at a two-sided significance level of 0.05.

If proportion not vaccinated in each subsequent season is 20%, the trial will have power of 0.954 and 0.929 to detect a risk reduction of 18% and 17%, respectively. On the other hand, if proportion not vaccinated in each subsequent season is 40%, the trial will have power of 0.929 and 0.896 to detect a risk reduction of 18% and 17%, respectively.

13.13 Level of Significance

The type I error probability for the primary endpoint will be 0.05, two sided. For all other analyses, two-sided p-values < 0.05 will be considered statistically significant with the number of comparisons made noted.

13.14 Stopping Rules for Termination of the Trial

The study will be monitored by a Data and Safety Monitoring Board (DSMB) as described in Section 13.19. At each meeting of the DSMB, the treatment groups will be compared with respect to the safety outcomes and the efficacy outcomes at the end of each enrolling season.

The vaccine will be administered once to each subject each season during the time vaccine is typically administered as standard of care. The end of season follow-up will extend to July 31 of each enrolling season. The efficacy of the vaccines will be assessed at the end of season follow-up as noted above. The DSMB may recommend stopping the study for other reasons, taking into account the efficacy and safety data from this trial and other studies, or concerns about study conduct.

In addition, early termination of this trial may occur because of a regulatory authority decision, withdrawal of study approval by clinical site IRBs, or investigational product safety problems. The NHLBI retains the right to discontinue the trial prior to the completion of enrollment of the targeted number of subjects, but will exercise these rights only for valid scientific or administrative reasons. The NHLBI will promptly notify the Executive Committee.

13.15 Spurious Data Procedures

Consistency checks and range checks will be built into the Clinical Trial Data Management System (CTDMS) OpenClinica. This will allow many errors to be identified and corrected at the time of data entry. Queries regarding any problems with data and trial conduct will be sent to site coordinators regularly throughout the course of the trial. Sites will also be monitored during the study as part of the trial quality control activities. Therefore, spurious data are expected to be infrequent.

The study monitoring report will indicate the number of subjects who have missing data on each study endpoint. For covariate-adjusted analyses, the number of subjects who have missing data on the covariates will be reported.

CONFIDENTIAL

Throughout the study, the rate, timing and reasons for subject withdrawal will be monitored. If necessary, retraining will take place and for-cause in-person site visits may be conducted by the Data Coordinating Center. The site may be barred from enrolling additional subjects to the study depending on the extent of the problems.

13.16 Deviation Reporting Procedures

Any modifications or deviations from the statistical analysis plan described in Sections 13.1 - 13.11 will be documented in the final Statistical Analysis Plan.

13.17 Subjects to be Included in Analyses

The mITT sample will be considered the primary analysis sample for all outcomes. All patients randomized will comprise the ITT sample. For the assessment of balance and reporting of adverse events and for the recurrent events analyses described in Section 13.5, we will use the ITT sample.

13.18 Measures to Minimize/Avoid Bias

13.18.1 Randomization

Randomization will be carried out using permuted blocks of random block size, stratified naturally by influenza season, balanced by clinical site.[50] Subjects will be randomized in a 1:1 ratio to high-dose or standard-dose vaccine. Subjects will remain on the same dose for subsequent influenza sessions. The clock is reset to zero each year for every subject, whether or not he or she continues with the study. That means that subsequent events are counted in the primary analysis only for persons who continue with the study, i.e., who are revaccinated per their original assignment. It also means that a person can contribute a new event to the primary endpoint for each year of participation in the trial. The trial will therefore employ a modified intention-to-treat (mITT) analysis in which subjects' clocks will be reset 2 weeks after influenza vaccination, and primary endpoint events will be counted until July 31 of that year. Events that occur after that point in persons who do not get revaccinated are unlikely to be related to the original vaccination. Although a subject's experiences from year to year may be correlated, for simplicity, each subject's contribution for each influenza season will be treated as independent.

Subjects will be randomized using Frontier Science and Technology Research Foundation's web-based the Statistical Registration and Randomization System (StaRRS) that is available 24/7 and only accessible to designated site personnel. StaRRS will confirm subject eligibility in a stepwise fashion as each protocol defined eligibility criteria is verified. Only when a subject meets all protocol-defined eligibility requirements will the system provide a randomization code in a blinded manner. A notification of successful randomization will appear within the application and will also be sent via e-mail to the clinical site. Detailed instructions can be found in the Statistical Registration and Randomization User Manuel.

13.18.2 Stratification

Randomization will not be stratified, except for the natural stratification by influenza season. Randomization will be balanced within clinical site, so that any site-specific characteristics will be approximately evenly balanced between the dose groups. While randomization does not ensure that any particular baseline characteristic will be balanced between treatment groups, due to a large sample

CONFIDENTIAL

size, not only the measured baseline characteristics but also unmeasured potential confounders are expected to be balanced equally between the two arms. Analysis of study findings will be post-stratified (during analysis) by age (≥ or < 65 years old), baseline cardiovascular risk group (AMI or HF), and influenza season, i.e. cohort year enrolled.

13.18.3 Masking

Subjects, site investigators and study personnel, persons performing surveillance, and data analysts will remain blind to the identity of the treatment from the time of randomization until database lock. Randomization data are kept strictly confidential until the time of unblinding, and will not be accessible by anyone involved in the study except as required by the Data and Safety Monitoring Board (DSMB), or in the case of subject emergencies, for which unblinding is deemed absolutely necessary. The identity of the vaccine doses will be concealed through packaging of the syringes by Biologics Inc.

13.19 Data and Safety Monitoring Board (DSMB)

A Data and Safety Monitoring Board (DSMB) appointed by the NHLBI will monitor the study. The DSMB consists of individuals with expertise in cardiovascular diseases, influenza, clinical trial methodology, biostatistics, and bioethics. The DSMB will review and approve the protocol prior to subject enrollment.

Early stopping guidelines are specified in Section 13.11 on Interim Analysis. The DSMB may recommend stopping the study early if the incidence of adverse events in the high-dose arm is greater than in the standard-dose arm or if the formal interim analysis shows clear efficacy results. Formal stopping boundaries are not proposed for other endpoints besides the primary endpoint. However, the DSMB may recommend stopping the study for other reasons, taking into account the efficacy and safety data from this trial and other studies, or concerns about study conduct.

The DSMB chair will be notified of any serious adverse events considered probably or definitely related to the study treatment. The DSMB reviews data on adverse events, adverse drug reactions, data quality, and study recruitment at regular intervals (every 6 months), and makes recommendations about study conduct to the Executive Committee.

13.20 Clinical Endpoints Committee

The primary objective of the Clinical Endpoints Committee (CEC) is consistent and unbiased review and classification of study endpoints throughout the course of the trial. The CEC, which will remain blinded to treatment assignment, will categorize and classify hospitalizations identified by surveillance in all subjects enrolled in INVESTED utilizing available medical records. Description of the endpoint operations and specific endpoint definitions are described in the CEC manual of operations and endpoint definitions document.

14.0 ETHICS AND PROTECTION OF HUMAN SUBJECTS

14.1 Ethical Standard

The trial will be conducted in accordance with the Declaration of Helsinki, Good Clinical Practice (GCP) guidelines, the rules and regulations of the Institutional Review Board(s), and applicable state and federal regulatory agency requirements and laws.

CONFIDENTIAL

14.2 Institutional Review Board (IRB)

The protocol, informed consent form(s), recruitment materials, and all subject materials will be submitted to the IRB for review and approval. Approval of both the protocol and the consent form must be obtained before any subject is enrolled. Any amendment to the protocol will require review and approval by the IRB before the changes are implemented in the study.

14.3 Informed Consent Process

Informed consent is a process that is initiated prior to the individual agreeing to participate in the study and continues throughout study participation. Extensive discussion of risks and possible benefits of study participation will be provided to subjects and their families, if applicable. A consent form describing in detail the study procedures and risks will be given to the subject. Consent forms will be IRB-approved, and the subject is required to read and review the document or have the document read to him or her. The investigator or designee will explain the research study to the subject and answer any questions that may arise. The subject will sign the informed consent document prior to any study-related assessments or procedures. Subjects will be given the opportunity to discuss the study with their surrogates or think about it prior to agreeing to participate. They may withdraw consent at any time throughout the course of the study. A copy of the signed informed consent document will be given to subjects for their records. The rights and welfare of the subjects will be protected by emphasizing to them that the quality of their clinical care will not be adversely affected if they decline to participate in this study. The consent process will be documented in the clinical or research record.

14.4 Exclusion of Women, Minorities and Children (Special Populations)

INVESTED aims to enroll a representative proportion of minority subjects. Children, pregnant women, prisoners, and institutionalized individuals will not be enrolled. Children are excluded because their heart disease phenotype differs from that of an adult, which could confound vaccine response results. Pregnant women are excluded because of regulations barring them from this type of research (i.e. the effects of a higher dose of influenza vaccine may have unknown risks to a fetus).

15.0 TRIAL MANAGMENT AND COMMUNICATION PLAN

15.1 Monitoring IRB Approvals

Each participating institution must provide for the review and approval this protocol and the associated informed consent documents and recruitment material by an appropriate IRB registered with the OHRP (for US sites). Any amendments to the protocol or consent materials must also be approved before they are placed into use. In the US, only institutions holding a current US Federal-wide Assurance or IRB registration number issued by OHRP may participate.

Site IRB approval letters will be collected by the CCC directly or by the designated representative from each network, who will forward information to the clinical coordinating center, and will be required for sites to be certified as ready to enroll subjects. The date of each IRB approval will be recorded and sites will need to inform the CCC about any changes to the IRB approval and all IRB renewals. In the event of a lapse in a sites IRB approval, the site will be responsible to obtain clearance from their IRB to continue to collect follow-up data on subjects enrolled, but no additional subjects can be enrolled from that site until full IRB approval is reinstated.

CONFIDENTIAL

15.2 Monitoring of Modifications

Modifications to the protocol can only be made by the executive committee in consultation with the NHLBI project office. Protocol modifications that affect study design or endpoints in any substantial manner may need to be reviewed by the NIH Protocol Review Committee or DSMB. Modifications to the protocol will be communicated to all sites through their respective networks or directly from the CCC if a site is not a member of one of the principal networks. When modifications of the protocol are made that require sites to resubmit the protocol to the local IRB, sites will be required to provide evidence of IRB re-approval before they can enroll subjects under the new protocol.

15.3 Problem Management

Issues that arise at sites regarding the conduct of the study must be reported by site personnel to their respective network or consortium leadership or to the study's principal investigators (CCC). Each network will be responsible for maintaining contact with sites and a representative of the overseeing network or clinical coordinating center will make contact with site personnel monthly during vaccination season and bimonthly subsequently. Medical monitors at the clinical coordinating center will be available to handle medical or trial related questions via telephone or email. Issues that cannot easily be resolved by study medical monitors will be discussed by the full executive committee at weekly meetings or on an ad hoc basis.

15.4 Communication

Study leadership (including Principal Investigators) will meet weekly or bi-weekly during the course of the trial. The full executive committee will meet via conference call at least bi-weekly throughout the course of the trial, and will include members of the NHLBI project office. Face to face meetings held at major cardiovascular meetings no fewer than biannually. Sites will be updated with study information by means of webinars, conducted prior to study enrollment and on an ad hoc basis as needed.

16.0 DATA COLLECTION AND QUALITY ASSURANCE

16.1 Data Management Responsibilities

The DCC will be responsible for implementing and maintaining quality control and quality assurance systems with written standard operating procedures (SOPs) to ensure that trial is conducted and data are generated, documented, and reported in compliance with the protocol, accepted standards of Good Clinical Practice (International Conference on Harmonization E6), and all applicable federal, state, provincial, and local laws, rules, and regulations relating to the conduct of the clinical trial.

During the trial, the DCC will utilize remote and risk-based monitoring to ensure that the protocol and good clinical practices (GCPs) are being followed as described in detail in a separate quality assurance document. Electronic monitoring will consist of reviewing and evaluating three separate components: conformance to IRB and consent form requirements with sampling of consents in 5% of subjects, source document data verification in 5% of subjects for entry criteria. The DCC will determine data accuracy using a number of statistical-based approaches to identify potential data errors. Source documents, such as documentation of entry criteria, may be queried and reviewed by members of the DCC and CCC to identify data irregularities and to confirm that the data recorded on the case report forms is accurate. Any site found to be Unacceptable or Acceptable/Needs Follow-up on monitoring is required to submit a written response and/or corrective action plan to the DCC within 21 days of the receipt of the final monitor findings. Sites that fail to meet the standards for acceptable performance will undergo follow-up action, which will be determined by the severity of the discrepancies and may include repeat on-site monitoring, probation, or suspension. For-cause monitoring will occur if

CONFIDENTIAL

irregularities are identified, and if necessary will be supplemented by site visits by members of the DCC or CCC or network staff. The Investigator and the clinical site will allow the monitors and appropriate regulatory authorities direct access to source documents to perform this verification.

The Clinical Site may be subject to review and/or inspection by the Institutional Review Board (IRB), the US Food and Drug Administration (FDA), Health Canada, and/or to quality assurance audits performed by the NHLBI or the DCC or CCC or network staff.

It is important that the Investigator(s) and the relevant clinical site personnel be available during the monitoring period and possible audits or inspections, and that sufficient time is devoted to the process.

16.2 Clinical Trial Data Management System

OpenClinica will be the clinical trial data management system (CTDMS) for the INVESTED trial. It is a web-based CTDMS thus it can be accessed from most locations in the world to support multi-center studies. OpenClinica is FDA 21 CFR Part 11 compliant and supports Good Clinical Practice (GCP) via differentiated user roles and privileges, password and user authentication security, electronic signatures, secure sockets layer (SSL) encryption, and a comprehensive auditing record and monitoring of access and data changes.

Based on the differentiated roles and privileges, subject data will only be available to the specific site which has enrolled the subject and entered data. All clinical data required by the protocol is entered using a unique subject assigned number and no personal identifying information is entered or stored other than dates of birth and service.

All data entered through OpenClinica will be stored on servers provided by OpenClinica, LLC which maintains all data in a SAS 70 Type II audit certification and meets ISO 17799 standards for information security. Access to OpenClinica on their servers is limited, via login credentials, to authorized users for the web interface only. Customers have no access to the server itself. All OpenClinica employees are granted access only to computer and networking areas necessary to perform their duties. Each installation is separate, and cannot be accessed from any other installation. Connection to a hosted instance is encrypted by means of secure socket layer. The application server and database server are secured via firewall, hardened to remove nonessential access credentials, and strong password compliance. Hosted systems are constantly monitored for latencies and intrusion.

16.3 Data Management Procedures

Data will be collected and entered into the CTDMS at each site participating in the trial. The CTDMS provides real-time field level validations, context sensitive help and automatic query generation. Data entry forms use browser based logic to enforce proper validations of all data fields and proper skip patterns within study data forms. Interim background data submittals prevent loss of data due to interruption of internet connections.

The CTDMS is programmed to validate data entry fields as the data are entered. Validations are question-by-question checks that give immediate feedback to help catch data entry errors, form completion errors, and out-of-range values. Reports of outstanding edits, generated upon completion of data entry, will enable continuous cleaning of data at each site. Detailed procedures are outlined in the Data Management Plan.

CONFIDENTIAL

If the DCC observes inconsistent data or patterns of protocol violations or missing data, site staff will be contacted to address the finding.

16.3.1 Data Use and Banking

After this research study is over and the main results have been published, a complete de-identified data set will be banked at the University of Wisconsin-Madison, Brigham and Women's Hospital, the University of Minnesota, and the University of Toronto including the following data: medical history and medications, vital signs, side effects or other health issues that occurred during the study, reasons for hospitalizations, health status, and results of immune testing for those in the substudy. Directly identifiable information will be removed and keys linking codes to subject identity will be destroyed, making data withdrawal impossible. The banked data will not be sent to researchers outside of UW-Madison, Brigham and Women's Hospital, University of Minnesota, and the University of Toronto, and will be used for future research about influenza and/or heart disease, but not necessarily about the interaction between the two. A de-identified dataset will also be sent to NIH BioLINCC and will be managed per NIH Data sharing policies.

16.4 Direct Access to Source Data

As noted above, source documents for verification of entry criteria may be queried by the DCC. The investigator will make available to the DCC source documents as requested. The verifications of CRF data will be made by direct review of source documents for a small percentage of subjects as described above. It may be necessary to have access to the complete medical record in some instances.

16.5 Specimen Collection Management

Ensuring the accuracy of blood specimens for this trial is paramount. The Serology Laboratories have quality control methods in place to ensure this accuracy. Aliquot tubes are linked, and tracked to allow tracing back to the original parent tube. The complete electronic chain of custody in place will allow the data management system to monitor and report on the critical processes involved in specimen collection, shipping and results. There is immediate feedback between the clinical site and the Serology Laboratories when a shipment of specimens is sent from the clinical site to either Brigham and Women's Hospital, or the Peter Munk Cardiac Centre.

16.6 Confidentiality of Data

By signing this protocol, the Investigator affirms to NHLBI that information furnished to the Investigator by the NHLBI will be maintained in confidence and such information will be divulged to the IRB or similar expert committee, affiliated institution, and employees only under an appropriate understanding of confidentiality with such board or committee. Data generated by this clinical trial will be considered confidential by the Investigator, except to the extent that it is included in a publication. Anonymized data from this trial will be made available according to NIH requirements.

16.7 Confidentiality of Subject Records

By signing the protocol, the Investigator agrees that the NHLBI, IRB, or Regulatory Agency representative may consult and/or copy study documents in order to verify CRF data. By signing the consent form, the subject agrees to this process. If study documents will be photocopied during the process of verifying CRF information, the subject will be identified by unique code only and full names and similar identifying information (such as medical record number or social security number) will be masked.

CONFIDENTIAL

The Clinical Site Investigators will ensure that the identity of subjects will be protected. All study records will be maintained in a secure fashion with access limited to essential study personnel only. All study documents submitted to the Data Coordinating Center will have identifiers removed other than age, dates of death, and service and subjects will be identified with a study-specific identification number only. The Clinical Site Investigators will maintain, in a secure location, an enrollment log that includes subject identifying information and links subjects to their study-specific identification number.

16.8 Protocol Deviations

A protocol deviation is any noncompliance with the clinical study protocol, Good Clinical Practice, or Manual of Procedures requirements. The noncompliance may be on the part of the subject, the investigator, or study staff.

Investigators may only implement a deviation from or a change to the protocol to eliminate an immediate hazard(s) to subjects without prior IRB approval.

All deviations from the protocol must be addressed in study subject source documents and promptly reported to Network Study Manager or Clinical Coordinating Center. As a result of deviations, corrective actions are to be developed by the study staff and implemented promptly.

17.0 PUBLICATION POLICY

Publication of the results of this trial will be governed by the policies and procedures developed by the Publications Committee, comprised of a subset of members from the Steering Committee. No results will be released publicly before completion of the final analysis regarding the primary endpoint of this study without the approval of the NHLBI, and the Executive Committee. Any presentation, abstract, or manuscript will be made available for review by the principal investigators and the NHLBI prior to submission.

This study will comply with the NIH Public Access Policy, which requires submission of final peer-reviewed journal manuscripts that arise from NIH funds to the digital archive *PubMed Central*.

18.0 REFERENCES

1. Smeeth L, Thomas SL, Hall AJ, Hubbard R, Farrington P, Vallance P. Risk of myocardial infarction and stroke after acute infection or vaccination. *The New England journal of medicine.* Dec 16 2004;351(25):2611-2618.

2. Warren-Gash C, Smeeth L, Hayward AC. Influenza as a trigger for acute myocardial infarction or death from cardiovascular disease: a systematic review. *Lancet Infect Dis.* Oct 2009;9(10):601-610.

3. Corrales-Medina VF, Musher DM, Shachkina S, Chirinos JA. Acute pneumonia and the cardiovascular system. *Lancet.* Feb 9 2013;381(9865):496-505.

4. Nichol KL, Nordin J, Mullooly J, Lask R, Fillbrandt K, Iwane M. Influenza vaccination and reduction in hospitalizations for cardiac disease and stroke among the elderly. *The New England journal of medicine.* Apr 3 2003;348(14):1322-1332.

5. Gwini SM, Coupland CA, Siriwardena AN. The effect of influenza vaccination on risk of acute myocardial infarction: self-controlled case-series study. *Vaccine.* Feb 1 2011;29(6):1145-1149.

6. Gurfinkel EP, Leon de la Fuente R, Mendiz O, Mautner B. Flu vaccination in acute coronary syndromes and planned percutaneous coronary interventions (FLUVACS) Study. *Eur Heart J.* Jan 2004;25(1):25-31.

7. Ciszewski A, Bilinska ZT, Brydak LB, et al. Influenza vaccination in secondary prevention from coronary ischaemic events in coronary artery disease: FLUCAD study. *Eur Heart J.* Jun 2008;29(11):1350-1358.

CONFIDENTIAL

8. Phrommintikul A, Kuanprasert S, Wongcharoen W, Kanjanavanit R, Chaiwarith R, Sukonthasarn A. Influenza vaccination reduces cardiovascular events in patients with acute coronary syndrome. *Eur Heart J.* Jul 2011;32(14):1730-1735.

9. Ajani UA, Ford ES, Mokdad AH. Examining the coverage of influenza vaccination among people with cardiovascular disease in the United States. *Am Heart J.* Feb 2005;149(2):254-259.

10. Kwong JC, Rosella LC, Johansen H. Trends in influenza vaccination in Canada, 1996/1997 to 2005. *Health reports / Statistics Canada, Canadian Centre for Health Information = Rapports sur la sante / Statistique Canada, Centre canadien d'information sur la sante.* Nov 2007;18(4):9-19.

11. McMurray JJV, Packer M, Gong J, et al. Pneumococcal/influenza vaccination, exercise prescription and disease-management programme participation in PARADIGM-HF (abstract). *European journal of heart failure.* May 1, 2013 2013;12(suppl 1):S10.

12. Udell JA, Zawi R, Bhatt DL, et al. Association between influenza vaccination and cardiovascular outcomes in high-risk patients: a meta-analysis. *JAMA.* Oct 23 2013;310(16):1711-1720.

13. McElhaney JE, Herre JM, Lawson ML, Cole SK, Burke BL, Hooton JW. Effect of congestive heart failure on humoral and ex vivo cellular immune responses to influenza vaccination in older adults. *Vaccine.* Jan 26 2004;22(5-6):681-688.

14. Vardeny O, Sweitzer NK, Detry MA, Moran JM, Johnson MR, Hayney MS. Decreased immune responses to influenza vaccination in patients with heart failure. *J Card Fail.* May 2009;15(4):368-373.

15. Vardeny O, Moran JJ, Sweitzer NK, Johnson MR, Hayney MS. Decreased T-cell responses to influenza vaccination in patients with heart failure. *Pharmacotherapy.* Jan;30(1):10-16.

16. Van Ermen A, Hermanson MP, Moran JM, Sweitzer NK, Johnson MR, Vardeny O. Double dose vs. standard dose influenza vaccination in patients with heart failure: a pilot study. *Eur J Heart Fail.* May 2013;15(5):560-564.

17. Keitel WA, Atmar RL, Cate TR, et al. Safety of high doses of influenza vaccine and effect on antibody responses in elderly persons. *Archives of internal medicine.* May 22 2006;166(10):1121-1127.

18. Falsey AR, Treanor JJ, Tornieporth N, Capellan J, Gorse GJ. Randomized, double-blind controlled phase 3 trial comparing the immunogenicity of high-dose and standard-dose influenza vaccine in adults 65 years of age and older. *The Journal of infectious diseases.* Jul 15 2009;200(2):172-180.

19. DiazGranados CA, Dunning AJ, Jordanov E, Landolfi V, Denis M, Talbot HK. High-dose trivalent influenza vaccine compared to standard dose vaccine in elderly adults: safety, immunogenicity and relative efficacy during the 2009-2010 season. *Vaccine.* Jan 30 2013;31(6):861-866.

20. DiazGranados CA, Robertson CA, Talbot HK, Landolfi V, Dunning AJ, Greenberg DP. Prevention of serious events in adults 65 years of age or older: A comparison between high-dose and standard-dose inactivated influenza vaccines. *Vaccine.* Sep 11 2015;33(38):4988-4993.

21. Couch RB, Winokur P, Brady R, et al. Safety and immunogenicity of a high dosage trivalent influenza vaccine among elderly subjects. *Vaccine.* Nov 1 2007;25(44):7656-7663.

22. Kaplan EL, Meier P. Nonparametric estimation from incomplete observations
53. 1958;282(457-481).

23. Cox DR. Regression models and life-tables. *J Roy Statist Soc.* 1972;34(2):187-220.

24. Frangakis CE, Rubin DB. Principal stratification in causal inference. *Biometrics.* Mar 2002;58(1):21-29.

25. Rosenbaum pr, Rubin DB, . The central role of the propensity score in observational studies for causal effects. *Biometrika.* 1983;70:41-55.

26. Xie J, Liu C. Adjusted Kaplan-Meier estimator and log-rank test with inverse probability of treatment weighting for survival data. *Stat Med.* Oct 30 2005;24(20):3089-3110.

27. Oakes D. Frailty models for multiple event times. In J. P. Klein and P.K. Goel eSASotAK, Netherlands, 1992.

28. Prentice rl, Cai j. Covariance and survivor function estimation using censored multivariate failure time data. *Biometrika.* 1992;79:495-512.

29. Fine jp, Gray rj. A proportional hazards model for the subdistribution of a competing risk. . *J Amer Statist Assoc.* 1999;94:496-509.

30. Anderson pk, Gill rd. Cox's regression model for counting processes: A large sample study. . *Ann Statist.* 1982;10:1100-1120.

CONFIDENTIAL

31. Prentice rl, Williams B, Peterson AV. On the regression analysis of multivariate failure time data. . *Biometrika.* 1981;68:373-379.
32. Ghosh D, Lin DY. Nonparametric analysis of recurrent events and death. *Biometrics.* Jun 2000;56(2):554-562.
33. Wei LJ, Lin DY. Regression analysis of multivariate incomplete failure time data by modeling marginal distributions. *J Amer Stat Assoc.* 1989;84:1065-1073.
34. Ghosh D, Lin DY. Semiparametric analysis of recurrent events data in the presence of dependent censoring. *Biometrics.* Dec 2003;59(4):877-885.
35. Daniel WW, ed *Applied Nonparametric Statistics.* 2nd ed: PWS-Kent; 1990.
36. Dunning AJ, DiazGranados CA, Voloshen T, Hu B, Landolfi VA, Talbot HK. Correlates of Protection against Influenza in the Elderly: Results from an Influenza Vaccine Efficacy Trial. *Clin Vaccine Immunol.* 2016;23(3):228-235.
37. Little RJ, D'Agostino R, Cohen ML, et al. The prevention and treatment of missing data in clinical trials. *The New England journal of medicine.* Oct 4 2012;367(14):1355-1360.
38. National Research Council. The Prevention and Treatment of Missing Data in Clinical Trials. Washington, DC: National Academies Press, 2010.
39. Lan KKG, Demets D. Discrete sequential boundaries for clinical trials. *Biometrika.* 1983;70:659-663.
40. O'Brien PC, Fleming TR. A multiple testing procedure for clinical trials. *Biometrics.* Sep 1979;35(3):549-556.
41. Pitt B, Remme W, Zannad F, et al. Eplerenone, a selective aldosterone blocker, in patients with left ventricular dysfunction after myocardial infarction. *The New England journal of medicine.* Apr 3 2003;348(14):1309-1321.
42. Fox K, Ford I, Steg PG, Tendera M, Ferrari R. Ivabradine for patients with stable coronary artery disease and left-ventricular systolic dysfunction (BEAUTIFUL): a randomised, double-blind, placebo-controlled trial. *Lancet.* Sep 6 2008;372(9641):807-816.
43. Swedberg K, Komajda M, Bohm M, et al. Ivabradine and outcomes in chronic heart failure (SHIFT): a randomised placebo-controlled study. *Lancet.* Sep 11 2010;376(9744):875-885.
44. Mega JL, Braunwald E, Wiviott SD, et al. Rivaroxaban in patients with a recent acute coronary syndrome. *The New England journal of medicine.* Jan 5 2012;366(1):9-19.
45. Steg PG, Bhatt DL, Wilson PW, et al. One-year cardiovascular event rates in outpatients with atherothrombosis. *JAMA.* Mar 21 2007;297(11):1197-1206.
46. Bhatt DL, Eagle KA, Ohman EM, et al. Comparative determinants of 4-year cardiovascular event rates in stable outpatients at risk of or with atherothrombosis. *JAMA.* Sep 22 2010;304(12):1350-1357.
47. McMurray JJ, Packer M, Desai AS, et al. Angiotensin-Neprilysin Inhibition versus Enalapril in Heart Failure. *The New England journal of medicine.* Aug 30 2014.
48. Packer M, McMurray JJ, Desai AS, et al. Angiotensin receptor neprilysin inhibition compared with enalapril on the risk of clinical progression in surviving patients with heart failure. *Circulation.* Jan 6 2015;131(1):54-61.
49. Bonaca MP, Bhatt DL, Cohen M, et al. Long-term use of ticagrelor in patients with prior myocardial infarction. *The New England journal of medicine.* May 7 2015;372(19):1791-1800.
50. Zelen M. The randomization and stratification of patients to clinical trials. *Journal of chronic diseases.* Sep 1974;27(7-8):365-375.
51. Bolger AP, Sharma R, Li W, et al. Neurohormonal activation and the chronic heart failure syndrome in adults with congenital heart disease. *Circulation.* 2002;106(1):92-99.
52. Diller GP, Dimopoulos K, Okonko D, et al. Exercise intolerance in adult congenital heart disease: comparative severity, correlates, and prognostic implication. *Circulation.* 2005;112(6):828-835.
53. Diller GP, Dimopoulos K, Okonko D, et al. Heart rate response during exercise predicts survival in adults with congenital heart disease. *J Am Coll Cardiol.* 2006;48(6):1250-1256.
54. Marelli AJ, Ionescu-Ittu R, Mackie AS, Guo L, Dendukuri N, Kaouache M. Lifetime prevalence of congenital heart disease in the general population from 2000 to 2010. *Circulation.* 2014;130(9):749-756.

CONFIDENTIAL

55. Kantor PF, Redington AN. Pathophysiology and management of heart failure in repaired congenital heart disease. *Heart Fail Clin.* 2010;6(4):497-506, ix.
56. Verheugt CL, Uiterwaal CS, van der Velde ET, et al. Mortality in adult congenital heart disease. *European heart journal.* 2010;31(10):1220-1229.
57. Harrison JL, Silversides CK, Oechslin EN, Kovacs AH. Healthcare needs of adults with congenital heart disease: study of the patient perspective. *J Cardiovasc Nurs.* 2011;26(6):497-503.
58. Mackie AS, Ionescu-Ittu R, Therrien J, Pilote L, Abrahamowicz M, Marelli AJ. Children and adults with congenital heart disease lost to follow-up: who and when? *Circulation.* 2009;120(4):302-309.
59. Warnes CA, Williams RG, Bashore TM, et al. ACC/AHA 2008 Guidelines for the Management of Adults with Congenital Heart Disease: a report of the American College of Cardiology/American Heart Association Task Force on Practice Guidelines (writing committee to develop guidelines on the management of adults with congenital heart disease). *Circulation.* 2008;118(23):e714-833.
60. Agarwal S, Sud K, Menon V. Nationwide Hospitalization Trends in Adult Congenital Heart Disease Across 2003-2012. *J Am Heart Assoc.* 2016;5(1).
61. Stout KK, Broberg CS, Book WM, et al. Chronic Heart Failure in Congenital Heart Disease: A Scientific Statement From the American Heart Association. *Circulation.* 2016;133(8):770-801.
62. Zomer AC, Vaartjes I, van der Velde ET, et al. Heart failure admissions in adults with congenital heart disease; risk factors and prognosis. *Int J Cardiol.* 2013;168(3):2487-2493.
63. Manes A, Palazzini M, Leci E, Bacchi Reggiani ML, Branzi A, Galie N. Current era survival of patients with pulmonary arterial hypertension associated with congenital heart disease: a comparison between clinical subgroups. *European heart journal.* 2014;35(11):716-724.
64. Norozi K, Wessel A, Alpers V, et al. Incidence and risk distribution of heart failure in adolescents and adults with congenital heart disease after cardiac surgery. *Am J Cardiol.* 2006;97(8):1238-1243.
65. Greutmann M, Tobler D, Kovacs AH, et al. Increasing mortality burden among adults with complex congenital heart disease. *Congenit Heart Dis.* 2015;10(2):117-127.
66. Griffiths ER, Kaza AK, Wyler von Ballmoos MC, et al. Evaluating failing Fontans for heart transplantation: predictors of death. *Ann Thorac Surg.* 2009;88(2):558-563; discussion 563-554.
67. Puley G, Siu S, Connelly M, et al. Arrhythmia and survival in patients >18 years of age after the mustard procedure for complete transposition of the great arteries. *Am J Cardiol.* 1999;83(7):1080-1084.
68. Graham TP, Jr., Bernard YD, Mellen BG, et al. Long-term outcome in congenitally corrected transposition of the great arteries: a multi-institutional study. *J Am Coll Cardiol.* 2000;36(1):255-261.
69. Zomer AC, Vaartjes I, Grobbee DE, Mulder BJ. Adult congenital heart disease: new challenges. *Int J Cardiol.* 2013;163(2):105-107.
70. Gatzoulis MA, Walters J, McLaughlin PR, Merchant N, Webb GD, Liu P. Late arrhythmia in adults with the mustard procedure for transposition of great arteries: a surrogate marker for right ventricular dysfunction? *Heart.* 2000;84(4):409-415.
71. Bouchardy J, Therrien J, Pilote L, et al. Atrial arrhythmias in adults with congenital heart disease. *Circulation.* 2009;120(17):1679-1686.
72. Janousek J, Paul T, Luhmer I, Wilken M, Hruda J, Kallfelz HC. Atrial baffle procedures for complete transposition of the great arteries: natural course of sinus node dysfunction and risk factors for dysrhythmias and sudden death. *Z Kardiol.* 1994;83(12):933-938.
73. Gatzoulis MA, Till JA, Somerville J, Redington AN. Mechanoelectrical interaction in tetralogy of Fallot. QRS prolongation relates to right ventricular size and predicts malignant ventricular arrhythmias and sudden death. *Circulation.* 1995;92(2):231-237.

CONFIDENTIAL

APPENDIX A

Contemporary Heart Failure and Myocardial Infarction Trials/Cohort Studies Cardiovascular Event Rates[40-48]

Trial/Registry	Cohort Treatment Arms	Eligibility	Endpoint	Event Rate (%)	Duration of Follow-up, months (Mn/Md)	Annualized Event Rate
SHIFT	HF Ivabradine Placebo	Age ≥18, stable NYHA II-IV any cause HF, LVEF ≤35%, sinus rhythm HR ≥70, hospitalized for HF w/in previous year, on stable background treatment	CV Death, HF Hosp.	28.7	22.9	15.0
			CV Death, HF Hosp., MI Hosp.	34.4		18.0
			CV Hosp.	30.0		15.7
BEAUTIFUL	Ischemic HF Ivabradine Placebo	Age ≥55 (or ≥18 if DM), established CAD, NYHA I-III HF, LVEF<40%, sinus rhythm, on stable background treatment	CV Death, HF Hosp., MI Hosp.	15.3	19	9.7
EPHESIS	Post MI LVD Eplerenone Placebo	Age ≥18, 3-14 d post-MI, NYHA II-III HF, LVEF≤40% (if DM NYHA I)	CV Death, CV Hosp.	26.7	16	20.0
PARADIGM-HF	HF Sacubitril/valsartan Enalapril	Age ≥18, NYHA II-IV any cause HF, LVEF ≤40%, HF Hosp. within prior year and elevated NPs	CV Death, HF Hosp.	26.5	27	11.8
			CV Hosp.	31.9		14.2
ATLAS-ACS 2	Post-ACS Rivaroxaban Placebo	Age ≥18, 7 d post-ACS (if <55y needed also DM or prior MI)	CV Death, MI, Stroke	10.7	13	9.9
REACH (registry)	Prior Ischemic Event	Age ≥45 with any prior ischemic event (CAD, Cerebrovasc, PAD)	CV Death, MI, Stroke, Ischemic Hosp. (not incl. HF)	29.9	48	7.5
	Recent (<1y) Ischemic Event	Age ≥45 with ischemic event within past year		36.0		9.0
PEGASUS	Prior MI Ticagrelor Placebo	Age ≥50, prior MI 1-3 yrs + 1 additional risk factor	CV Death, MI, Stroke	9.0	36	3.3

CONFIDENTIAL

APPENDIX B

INVESTED-ACHD Sub-Study

Principal Investigator: Payam Dehghani, MD

We will prospectively assess a 500-patient cohort comprised of adults with congenital heart disease (ACHD) who are enrolled in the INVESTED clinical trial to test the hypothesis that high dose vaccine will reduce the composite of death or cardiopulmonary hospitalizations compared with standard dose. A common concern is whether higher dose vaccination confers a higher risk for adverse events.

Total duration of subject participation will be up to 3 influenza seasons. This sub study is supported by Prairie Vascular Research Inc.

BIOLOGICAL PLAUSIBILITY

The rationale behind INVESTED is that high-risk patients have blunted immune response to routine vaccination and may need higher doses. One cohort of high-risk patients are ACHD patients who have documented elevation in neurohormonal activation[51], decreased MVO2[52], and more pronounced chronotropic incompetence[53]. They ultimately succumb to heart failure due to structural abnormalities leading to valvular heart disease or chamber enlargement, systemic sub-pulmonic ventricles, residual shunts, atrial and or ventricular arrhythmias, pulmonary hypertension, and acquired coronary artery disease. We believe the above mechanisms lead to a blunted immune response and this sub study is designed to test the hypothesis that the ACHD population will benefit from the high-dose influenza vaccine.

POLICY AND PUBLIC HEALTH IMPLICATION

With advances in diagnosis and therapy, a growing number of children with congenital heart disease are becoming adults, such that as of 2010, patients with ACHD outnumber newborns with congenital heart disease[54]. The growing number and aging of ACHD patients has led to an increase in hospitalizations and presentation of heart failure over the last decade[55] with registry data suggesting that ACHD admission for heart failure confers a five-fold higher risk of death compared to those not admitted[56]. ACHD patients do not readily report symptoms and may present late during the course of their illness. What amplifies the burden of disease in this population is that they are often marginalized, have higher psychosocial stresses[57], and are lost to follow-up while transitioning to adult care[58]. This leads to poor access to evidence- based preventive strategies such as vaccination, primary care visits and follow-up in tertiary care centers with expertise in ACHD care. Given the prevalence of heart failure in this population, ACHD patients should be regarded as a high priority in need of influenza vaccination. ACHD patients, therefore, serve as an ideal population to study the effect of an intervention such as influenza vaccination as (1) most will have sub-clinical and/or clinical congestive heart failure (2) most will have historically poor access to preventive strategies such as vaccination. This trial, if positive, has the potential to substantially impact a major

CONFIDENTIAL

population attributable CV risk, change practice, and inform health policy by boosting utilization of influenza vaccination

STUDY OBJECTIVES

PRIMARY OBJECTIVE

To test the hypothesis that high dose (4x) trivalent influenza vaccine will reduce the composite of death or cardiopulmonary hospitalizations compared with standard dose quadrivalent influenza vaccine in the ACHD subgroup of the INVESTED trial.

SECONDARY OBJECTIVES

1. To test the hypothesis that compared to non-ACHD patients enrolled in INVESTED trial, ACHD patients will have a higher composite endpoint of death or cardiopulmonary hospitalizations.
2. To compare the effect of high-dose influenza vaccine versus standard-dose vaccine on time to first occurrence of cardiovascular death or cardiovascular hospitalization in the ACHD subgroup of the INVESTED trial.

To test the hypothesis that compared to non-ACHD patients enrolled in INVESTED trial, ACHD patients will have a lower rate of appropriate influenza vaccination in the season(s) preceding their enrolment.

REGISTRATION PROCEDURES

Patients must be randomized to the parent INVESTED Protocol, in order to be registered to this ACHD sub study. Registration can be completed as the patient is randomized to the INVESTED Trial, or concurrent with registration for returning INVESTED years 2 or 3.

Patients previously randomized to and participating in the INVESTED trial may be retroactively entered on this sub study concurrent with registration for Returning years 2 and 3.

Patients entered retroactively on this sub study will have previously given permission to be enrolled in the INVESTED protocol which informs them that their medical records will be reviewed, and questions about medical history and medications will be asked. The consent form indicates that health information will be accessed, collected and sent to the data coordinating center for research purposes. It is at the discretion of the individual IRB whether additional consent must be obtained for these patients.

Patients will be registered using Frontier Science's web-based Statistical Registration and Randomization system.

STUDY PROCEDURES

Study Procedures in the INVESTED protocol will be followed. No additional visits or procedures are required for this ACHD sub study.

CONFIDENTIAL

The ACHD case report form should be completed for each enrolled patient within 1 week following study registration.

ASSESSMENT SCHEDULE AND RECORDS TO BE KEPT

The ACHD Case Report Form (CRF), should be completed in OpenClinica for each enrolled patient within 1 week of study registration.

The focus of the ACHD CRF is to identify predictors of hospitalization, morbidity and mortality. We recognize that filling out the ACHD CRF on ACHD patients may be a daunting task as there may be lack of clarity about patient's lesion characteristics, operation(s) performed, and the allied health professional filling out the form may not be necessarily well-versed in ACHD terminology. If necessary research staff should obtain assistance from ACHD professionals following these patients.

The CRF aims to identify the lesion characteristics. As per 2008 Guidelines it stratifies lesion characteristics into simple, moderate and severe complexity[59]. Although general re-admission rates for patients with simple defects have increased significantly over the last decade, patients with complex defects have higher morbidity and mortality[60,61]. In addition to the type of lesion, following associated features are also predictors of increased morbidity and mortality[61] pulmonary hypertension[62,63], residual left and right outflow obstruction[64], cyanosis[65], Fontan circulation[65,66], uni-ventricular physiology[65], systemic right ventricular function[64,67,68], and decreased sub-pulmonic ventricular function[64]. In addition, the age that the surgery was first performed and number of subsequent surgical interventions are important predictors of mortality and readmissions[69,70].

Items on the CRF attempt to identify a patient's history of arrhythmias and baseline EKG characteristics, as well as intervention history. Atrial arrhythmias occur in 15% of adults with congenital heart disease and are associated with a doubling of the risk of adverse events[71]. Non-sinus rhythm and/or heart block are also poor prognosticators in subsets of ACHD patients[70,72] as are readily identifiable ECG characteristics such as QRS duration and QTc interval[70,73]. Section C collects EKG data. Section D of the CRF addresses whether care delivered to the patient has been delivered by someone and/or a center with expertise in ACHD and in a consistent manner. Expertise in care and regular surveillance of subtypes of congenital heart effect outcome and are guideline recommendations[59].

~~OUTCOME MEASURES~~ PRIMARY ENDPOINT

The primary endpoint of the present study is the time to first occurrence of death or cardiopulmonary hospitalization within each influenza season in the ACHD population.

CONFIDENTIAL CONFIDENTIAL

STATISTICAL CONSIDERATIONS

<u>Sample Size Calculation and Statistical Analysis</u>

The enrollment target will be 500 ACHD patients in the entire trial. There is no available data in the literature to help estimate risk reduction of composite endpoint in ACHD population.

Therefore, this will be an exploratory study to determine if there is effect modification by baseline presence of ACHD.

Demographic and medical characteristics of the ACHD and non-ACHD sample will be summarized with descriptive statistics such as mean (standard deviations) or median (IQR) for quantitative outcomes and frequency (percentage) for qualitative outcomes. These findings will also be summarized graphically comparing the two samples using two-sample tests. Clinical outcomes will be compared between those who have a diagnosis of ACHD and those who do not using log-rank tests. Cox proportional hazards regression analysis and confidence intervals will be performed for the primary endpoint of all-cause death or cardiopulmonary hospitalization. All tests will be two-tailed with a significance level of 0.05.

Appendix C

In the US, subjects who consent to participate in linkage of Medicare and Medicaid beneficiary data will have their health plan beneficiary numbers collected during the screening/baseline visit. Study sites will directly submit these subject identifiers, along with name, date of birth, and study ID, to the RedCap server that is located within the Partners Healthcare firewall. INVESTED trial data (including subjects' study ID) will be also uploaded by the Trial Data Coordination Center to RedCap server. The Brigham and Women's Department of Pharmacoepidemiology and Pharmacoeconomics, which manages the RedCap server, will directly submit the file containing the names and dates of birth of trial participants to the Centers for Medicare & Medicaid Services (CMS) Research Data Assistance Center (ResDAC) to obtain the following information: hospitalizations, emergency room visits, outpatient visits, or medication use that occur after or outside of the trial. The Brigham and Women's Department of Pharmacoepidemiology and Pharmacoeconomics will conduct the linkage to trial data. After linkage, the linked data will be stripped of all direct patient identifiers and a limited data file will be used by investigators in the Brigham and Women's Department of Pharmacoepidemiology and Pharmacoeconomics to conduct the data analyses. The data will be collected for up to 10 years post Year 1 baseline visit.

For subjects consenting to insurance linkage, their electronic Centers for Medicare & Medicaid Services (CMS) claims data will be obtained through (ResDAC) for linkage to the trial data. The linkage requires using Medicare's research identifiable files (RIF) that contain beneficiary-level protected health information (PHI). Using ResDAC's crosswalk file system, a file containing direct identifiers of the INVESTED participants will be submitted to the Medicare data finder to perform deterministic linkage. The system will extract claims data for consented subjects who are Medicare beneficiaries. (**Figure 3**).

CONFIDENTIAL

.

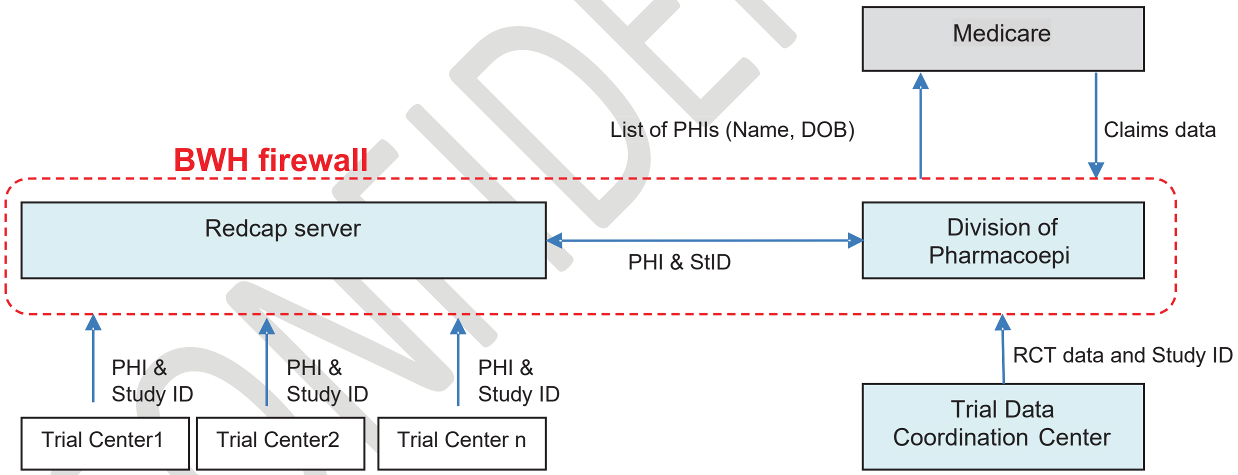

CONFIDENTIAL

Figure 3. Data linkage process
