# Supplementary material for: Influenza Vaccine Immune Response in Patients With High-Risk Cardiovascular Disease: A Secondary Analysis of the INVESTED Randomized Clinical Trial
Source: JAMA Cardiol. 2024 Apr 7;9(6):574–81. doi: 10.1001/jamacardio.2024.0468 (PMC11000133; doi:10.1001/jamacardio.2024.0468)
Supplement: Supplement 2. — eTable 1. Baseline Characteristics by Immune Response Sub-Study Participation Status eTable 2. Baseline Characteristics According to Seroconversion Status by Randomized Treatment eTable 3. Seroconversion by Total Number of Eligibility Risk Factors eTable 4. Primary and Key Secondary Outcomes by Seroconversion Status eTable 5. Cardiopulmonary Hospitalization or All-Cause Death According to Ordinate Levels of Seroprotection after 4-Weeks eTable 6. Cardiopulmonary Hospitalization or All-Cause Death According to Mean Log-Titer Levels at 4 Weeks as a Continuous Measure eFigure. Multivariate Predictors of Seroconversion Status eMethods. Vaccine Formulations by Randomized Treatment According to Influenza Season [file jamacardiol-e240468-s002.pdf]

Supplemental Online Content

Peikert A, Claggett BL, Udell JA, et al. Influenza vaccine immune response in patients with high-risk cardiovascular disease: a secondary analysis of the INVESTED randomized clinical trial. *JAMA Cardiol*. Published online April 7, 2024.
doi:10.1001/jamacardio.2024.0468

eTable 1. Baseline Characteristics by Immune Response Sub-Study Participation Status
eTable 2. Baseline Characteristics According to Seroconversion Status by Randomized Treatment
eTable 3. Seroconversion by Total Number of Eligibility Risk Factors
eTable 4. Primary and Key Secondary Outcomes by Seroconversion Status
eTable 5. Cardiopulmonary Hospitalization or All-Cause Death According to Ordinate Levels of Seroprotection after 4-Weeks
eTable 6. Cardiopulmonary Hospitalization or All-Cause Death According to Mean Log-Titer Levels at 4 Weeks as a Continuous Measure
eFigure. Multivariate Predictors of Seroconversion Status
eMethods. Vaccine Formulations by Randomized Treatment According to Influenza Season

This supplemental material has been provided by the authors to give readers additional information about their work.

© 2024 Peikert A et al. *JAMA Cardiology.*

eTable 1. Baseline Characteristics by Immune Response Sub-Study Participation Status.

Characteristic	Overall Population (n=5260)	Participants not included in Immune Response Sub-study (n=4602)	Participants included in Immune Response Sub-Study (n=658)	*P*-value
High-dose vaccine	2630 (50.0%)	2308 (50.2%)	322 (48.9%)	0.56
Randomization Year				0.66
1	494 (9.4 %)	426 (9.3 %)	68 (10.3%)	
2	2502 (47.6%)	2190 (47.6%)	312 (47.4%)	
3	2264 (43.0%)	1986 (43.2%)	278 (42.2%)	
Country				
Canada	1676 (31.9%)	1376 (29.9%)	300 (45.6%)	<0.001
United States	3584 (68.1%)	3226 (70.1%)	358 (54.4%)	<0.001
Age	65.5 ± 12.6	65.4 ± 12.7	66.2 ± 11.4	0.12
Female	1473 (28.0%)	1322 (28.7%)	151 (22.9%)	0.002
Race[a]				<0.001
Asian	155 (2.9 %)	130 (2.8 %)	25 (3.8 %)	
Black	784 (14.9%)	742 (16.1%)	42 (6.4 %)	
First Nation / American Indian	49 (0.9 %)	48 (1.0 %)	1 (0.2 %)	
Other	169 (3.2 %)	159 (3.5 %)	10 (1.5 %)	
White	4103 (78.0%)	3523 (76.6%)	580 (88.1%)	
Ethnicity[a]				0.005
Hispanic/Latino	517 (9.8 %)	473 (10.3%)	44 (6.7 %)	
Non-Hispanic/Latino	4683 (89.0%)	4073 (88.5%)	610 (92.7%)	
Other	60 (1.1 %)	56 (1.2 %)	4 (0.6 %)	
Ejection Fraction	42.2 ± 16.2	41.7 ± 16.3	45.4 ± 14.9	<0.001
New York Heart Association Functional Classification				<0.001
1	543 (17.2%)	453 (16.1%)	90 (26.1%)	
2	1584 (50.1%)	1415 (50.2%)	169 (49.0%)	
3	931 (29.4%)	853 (30.3%)	78 (22.6%)	
4	104 (3.3 %)	96 (3.4 %)	8 (2.3 %)	
BMI	30.9 ± 7.4	30.9 ± 7.5	31.0 ± 7.1	0.64
Qualifying Event				
Heart Failure	3289 (62.7%)	2941 (64.1%)	348 (52.9%)	<0.001
Myocardial Infarction	1960 (37.3%)	1650 (35.9%)	310 (47.1%)	<0.001

© 2024 Peikert A et al. *JAMA Cardiology.*

Eligibility Risk Factors[b]				
Age >=65'	2987 (56.9%)	2606 (56.8%)	381 (57.9%)	0.58
Current BMI >=30'	2551 (48.6%)	2221 (48.4%)	330 (50.2%)	0.39
Left Ventricular Ejection Fraction <40'	2208 (42.1%)	1960 (42.7%)	248 (37.7%)	0.015
Type I or type II diabetes mellitus'	1950 (37.1%)	1721 (37.5%)	229 (34.8%)	0.18
History of renal impairment'	1587 (30.2%)	1408 (30.7%)	179 (27.2%)	0.07
Current tobacco smoker'	902 (17.2%)	805 (17.5%)	97 (14.7%)	0.08
Prior Heart Failure'	903 (17.2%)	809 (17.6%)	94 (14.3%)	0.034
Prior Myocardial Infarction'	745 (14.2%)	616 (13.4%)	129 (19.6%)	<0.001
History of ischemic stroke'	433 (8.2 %)	385 (8.4 %)	48 (7.3 %)	0.34
History of peripheral artery disease'	232 (4.4 %)	205 (4.5 %)	27 (4.1 %)	0.67
Other Medical History[c]				
Hypertension	4046 (77.1%)	3556 (77.5%)	490 (74.5%)	0.09
Dyslipidemia	3616 (68.9%)	3130 (68.2%)	486 (73.9%)	0.003
Atrial Fibrillation	1725 (32.9%)	1518 (33.1%)	207 (31.5%)	0.41
Coronary Artery Bypass Graft	1040 (19.8%)	900 (19.6%)	140 (21.3%)	0.31
Percutaneous Coronary Intervention	2162 (41.2%)	1845 (40.2%)	317 (48.2%)	<0.001
ICD	956 (18.2%)	860 (18.7%)	96 (14.6%)	0.010
Asthma	602 (11.5%)	519 (11.3%)	83 (12.6%)	0.32
Chronic Obstructive Pulmonary Disease	1006 (19.2%)	885 (19.3%)	121 (18.4%)	0.59
Prior Influenza Vaccine	4060 (77.2%)	3515 (76.4%)	545 (82.8%)	<0.001
Prior Influenza Infection	1132 (21.5%)	956 (20.8%)	176 (26.7%)	<0.001
Maintenance Medication (Index Event MI)				
Aspirin	1785 (91.1%)	1512 (91.6%)	273 (88.1%)	0.043
HMG-CoA Reductase Inhibitors	1835 (93.6%)	1545 (93.6%)	290 (93.5%)	0.95
Beta Adrenergic Blocker	1679 (85.7%)	1419 (86.0%)	260 (83.9%)	0.33
Maintenance Medication (Index Event HF)				
Beta Adrenergic Blockers	2779 (84.5%)	2483 (84.4%)	296 (85.1%)	0.76
Diuretic	2607 (79.3%)	2326 (79.1%)	281 (80.7%)	0.47
Mineralocorticoid Receptor Antagonist	1118 (34.0%)	1003 (34.1%)	115 (33.0%)	0.69
Digoxin	314 (9.5 %)	285 (9.7 %)	29 (8.3 %)	0.42
ACE Inhibitor or ARB or ARNI	2197 (66.8%)	1964 (66.8%)	233 (67.0%)	0.95

Data are presented as mean ± SD and numbers (%). Analyses were based on the total number of participants. *P*-values are reported for differences between participants who were and those who were not included in the immune response sub-study. ACE indicates angiotensin-converting enzyme; ARB, angiotensin II receptor blocker; ARNI, angiotensin receptor neprilysin inhibitor; BMI, body mass index; LVEF, left ventricular ejection fraction; MI, myocardial infarction and HF, heart failure.

[a] Race and ethnicity were collected via patient self-report.

© 2024 Peikert A et al. *JAMA Cardiology.*

[b] Total may be greater than 100% because of multiple risk factors per participant.
[c] Medical history was collected via patient self-report and chart review.

© 2024 Peikert A et al. *JAMA Cardiology*.

eTable 2. Baseline Characteristics According to Seroconversion Status by Randomized Treatment.

Characteristic	Standard-Dose Quadrivalent Vaccine (n=336)			High-Dose Trivalent Vaccine (n=322)		
	No Seroconversion (n=109)	Seroconversion (n=227)	*P*-value	No Seroconversion (n=78)	Seroconversion (n=244)	*P*-value
Randomization Year			.10			.022
1	15 (13.8%)	20 (8.8 %)		13 (16.7%)	20 (8.2 %)	
2	56 (51.4%)	102 (44.9%)		41 (52.6%)	113 (46.3%)	
3	38 (34.9%)	105 (46.3%)		24 (30.8%)	111 (45.5%)	
Country			.64			.011
Canada	46 (42.2%)	102 (44.9%)		27 (34.6%)	125 (51.2%)	
United States	63 (57.8%)	125 (55.1%)		51 (65.4%)	119 (48.8%)	
Age	67.3 ± 10.3	66.4 ± 11.2	.51	66.9 ± 13.8	65.3 ± 11.3	.30
Female	24 (22.0%)	61 (26.9%)	.34	12 (15.4%)	54 (22.1%)	.20
Race[a]			.52			.57
Asian	4 (3.7 %)	7 (3.1 %)		1 (1.3 %)	13 (5.3 %)	
Black	6 (5.5 %)	17 (7.5 %)		4 (5.1 %)	15 (6.1 %)	
First Nation / American Indian	0 (0.0 %)	0 (0.0 %)		0 (0.0 %)	1 (0.4 %)	
Other	3 (2.8 %)	2 (0.9 %)		1 (1.3 %)	4 (1.6 %)	
White	96 (88.1%)	201 (88.5%)		72 (92.3%)	211 (86.5%)	
Ethnicity[a]			.50			.10
Hispanic/Latino	10 (9.2 %)	13 (5.7 %)		9 (11.5%)	12 (4.9 %)	
Non-Hispanic/Latino	98 (89.9%)	212 (93.4%)		69 (88.5%)	231 (94.7%)	
Other	1 (0.9 %)	2 (0.9 %)		0 (0.0 %)	1 (0.4 %)	
Ejection Fraction	42.9 ± 14.9	44.8 ± 14.5	.31	45.5 ± 14.7	47.2 ± 15.1	.43
New York Heart Association Functional Classification			.67			.37
1	19 (30.2%)	25 (22.3%)		15 (33.3%)	31 (24.8%)	
2	28 (44.4%)	53 (47.3%)		24 (53.3%)	64 (51.2%)	
3	14 (22.2%)	31 (27.7%)		5 (11.1%)	28 (22.4%)	
4	2 (3.2 %)	3 (2.7 %)		1 (2.2 %)	2 (1.6 %)	
BMI	31.0 ± 6.9	31.6 ± 7.4	.46	30.4 ± 6.2	30.7 ± 7.2	.73
Qualifying Event			.14			.32
Heart Failure	64 (58.7%)	114 (50.2%)		45 (57.7%)	125 (51.2%)	

© 2024 Peikert A et al. *JAMA Cardiology.*

Myocardial Infarction	45 (41.3%)	113 (49.8%)		33 (42.3%)	119 (48.8%)	
Eligibility Risk Factors[b]						
Age >=65'	68 (62.4%)	134 (59.0%)	.56	48 (61.5%)	131 (53.7%)	.22
Current BMI >=30	57 (52.3%)	122 (53.7%)	.80	32 (41.0%)	119 (48.8%)	.23
Left Ventricular Ejection Fraction <40	47 (43.1%)	84 (37.0%)	.28	32 (41.0%)	85 (34.8%)	.32
Type I or type II diabetes mellitus'	49 (45.0%)	73 (32.2%)	.022	21 (26.9%)	86 (35.2%)	.17
History of renal impairment'	28 (25.7%)	61 (26.9%)	.82	21 (26.9%)	69 (28.3%)	.82
Current tobacco smoker'	15 (13.8%)	32 (14.1%)	.93	8 (10.3%)	42 (17.2%)	.14
Prior Heart Failure'	15 (13.8%)	29 (12.8%)	.80	10 (12.8%)	40 (16.4%)	.45
Prior Myocardial Infarction'	28 (25.7%)	43 (18.9%)	.16	15 (19.2%)	43 (17.6%)	.75
History of ischemic stroke'	9 (8.3 %)	19 (8.4 %)	.97	8 (10.3%)	12 (4.9 %)	.09
History of peripheral artery disease'	9 (8.3 %)	6 (2.6 %)	.020	3 (3.8 %)	9 (3.7 %)	.95
Other Medical History[c]						
Hypertension	83 (76.1%)	168 (74.0%)	.67	60 (76.9%)	179 (73.4%)	.53
Dyslipidemia	79 (72.5%)	173 (76.2%)	.46	62 (79.5%)	172 (70.5%)	.12
Atrial Fibrillation	36 (33.0%)	69 (30.4%)	.63	33 (42.3%)	69 (28.3%)	.020
Coronary Artery Bypass Graft	32 (29.4%)	50 (22.0%)	.14	13 (16.7%)	45 (18.4%)	.72
Percutaneous Coronary Intervention	53 (48.6%)	112 (49.3%)	.90	40 (51.3%)	112 (45.9%)	.41
ICD	21 (19.3%)	29 (12.8%)	.12	13 (16.7%)	33 (13.5%)	.49
Asthma	14 (12.8%)	27 (11.9%)	.80	13 (16.7%)	29 (11.9%)	.28
Chronic Obstructive Pulmonary Disease	19 (17.4%)	51 (22.5%)	.29	12 (15.4%)	39 (16.0%)	.90
Prior Influenza Vaccine	102 (93.6%)	182 (80.2%)	.001	72 (92.3%)	189 (77.5%)	.004
Prior Influenza Infection	29 (26.6%)	65 (28.6%)	.70	21 (26.9%)	61 (25.0%)	.73
Maintenance Medication (Index Event MI)						
Aspirin	39 (86.7%)	100 (88.5%)	.75	27 (81.8%)	107 (89.9%)	.20
HMG-CoA Reductase Inhibitors	42 (93.3%)	106 (93.8%)	.91	30 (90.9%)	112 (94.1%)	.51
Beta Adrenergic Blocker	40 (88.9%)	92 (81.4%)	.25	29 (87.9%)	99 (83.2%)	.51
Maintenance Medication (Index Event HF)						

© 2024 Peikert A et al. *JAMA Cardiology.*

Beta Adrenergic Blockers	56 (87.5%)	100 (87.7%)	.97	36 (80.0%)	104 (83.2%)	.63
Diuretic	49 (76.6%)	101 (88.6%)	.034	34 (75.6%)	97 (77.6%)	.78
Mineralocorticoid Receptor Antagonist	20 (31.2%)	41 (36.0%)	.52	16 (35.6%)	38 (30.4%)	.52
Digoxin	5 (7.8 %)	7 (6.1 %)	.67	6 (13.3%)	11 (8.8 %)	.38
ACE Inhibitor or ARB or ARNI	44 (68.8%)	82 (71.9%)	.65	33 (73.3%)	74 (59.2%)	.09

Data are presented as mean ± SD and numbers (%). Seroconversion was defined as a ≥4-fold titer increase between baseline and 4-weeks for any of the antigens including A/H2N3 Hong Kong, A/H1N1 Michigan, A/H2N3 Singapore, B/Maryland, B/Brisbane, and B/Phuket. Analyses were based on the total number of participants. *P*-values are reported for differences between participants who did and those who did not achieve seroconversion. ACE indicates angiotensin-converting enzyme; ARB, angiotensin II receptor blocker; ARNI, angiotensin receptor neprilysin inhibitor; BMI, body mass index; LVEF, left ventricular ejection fraction; MI, myocardial infarction and HF, heart failure.

[a] Race and ethnicity were collected via patient self-report.

[b] Total may be greater than 100% because of multiple risk factors per participant.

[c] Medical history was collected via patient self-report and chart review.

© 2024 Peikert A et al. *JAMA Cardiology.*

eTable 3. Seroconversion by Total Number of Eligibility Risk Factors.

Total No. of Eligibility Risk Factors per Participant	Proportion of Participants[a] without Seroconversion	Proportion of Participants[a] with Seroconversion	Odds Ratio (95% CI)	*P*-value
1	53/175 (30.3%)	122/175 (69.7%)	Ref.	-
2	64/224 (28.6%)	160/224 (71.4%)	1.08 (0.77 to 1.53)	.64
3	59/208 (28.4%)	149/208 (71.6%)	1.12 (0.79 to 1.59)	.52
>=4	61/185 (33.0%)	124/185 (67.0%)	0.88 (0.61 to 1.26)	.48

Seroconversion was defined as a ≥4-fold titer increase between baseline and 4-weeks for any of the antigens including A/H2N3 Hong Kong, A/H1N1 Michigan, A/H2N3 Singapore, B/Maryland, B/Brisbane, or B/Phuket. Eligibility risk factors included age > 65 years, current or prior EF < 40%, diabetes, BMI >=30, history of chronic kidney disease (defined as estimated glomerular filtration rate ≤60 for at least 2 readings in the past year), ischemic stroke, peripheral artery disease, and current tobacco use. Estimated odds ratios and 95% confidence for the likelihood of seroconversion were derived from logistic regression models. Models were adjusted for country.

[a] Based on the total number of participant-seasons combined.

© 2024 Peikert A et al. *JAMA Cardiology.*

eTable 4. Primary and Key Secondary Outcomes by Seroconversion Status

Outcome	Events in Participants without Seroconversion (n= 237 Participant Seasons)	Events in Participants with Seroconversion (n= 555 Participant Seasons)	Hazard Ratio (95% CI)	*P*-value
**Primary**				
First cardiopulmonary hospitalization or all-cause death[a]	50 (21.1%)	120 (21.6%)	1.09 (0.79 to 1.53)	.59
**Secondary**				
Cardiopulmonary hospitalization[a]	47 (19.8%)	110 (19.8%)	1.06 (0.75 to 1.50)	.75
Cardiovascular hospitalization[a]	42 (17.7%)	91 (16.4%)	0.96 (0.66 to 1.39)	.82
MI hospitalization[a]	4 (1.7%)	10 (1.8%)	1.10 (0.33 to 3.62)	.88
Heart Failure hospitalization[a]	15 (6.3%)	39 (7.0%)	1.17 (0.63 to 2.15)	.62
Pulmonary hospitalization[a]	8 (3.4%)	13 (2.3%)	0.78 (0.32 to 1.86)	.57
Flu/Pneumonia hospitalization[a]	1 (0.4%)	5 (0.9%)	2.25 (0.28 to 18.32)	.45
Non-Cardiopulmonary hospitalization[a]	33 (13.9%)	61 (11.0%)	0.85 (0.55 to 1.33)	.48
Hospitalization for any cause[a]	66 (27.8%)	147 (26.5%)	1.01 (0.75 to 1.36)	.94
All-cause death[b]	12 (9.3%)	24 (6.3%)	0.80 (0.40 to 1.60)	.52

Seroconversion was defined as a ≥4-fold titer increase between baseline and 4-weeks for any of the antigens A/H2N3 Hong Kong, A/H1N1 Michigan, A/H2N3 Singapore, B/Maryland, B/Brisbane, or B/Phuket. Models were adjusted for country.
[a] For each participant season, based on the number of all participant-seasons combined.
[b] Based on the number of participants, across all seasons.

© 2024 Peikert A et al. *JAMA Cardiology.*

eTable 5. Cardiopulmonary Hospitalization or All-Cause Death According to Ordinate Levels of Seroprotection after 4-Weeks

Antigen Type	Events in Participants with Titer <40, No./Total No. (%)	Events in Participants with Titer 40-79, No./Total No. (%)	Events in Participants with Titer 80-159, No./Total No. (%)	Events in Participants with Titer 160-319, No./Total No. (%)	Events in Participants with Titer ≥320, No./Total No. (%)	Hazard Ratio (95% CI	*P*-value
A/H2N3 Hong Kong	2/9 (22.2%)	4/16 (25.0%)	3/16 (18.8%)	6/42 (14.3%)	50/207 (24.2%)	1.05 (0.81 to 1.36)	0.72
A/H1N1 Michigan (2017/2018)	3/7 (42.9%)	2/11 (18.2%)	6/33 (18.2%)	9/37 (24.3%)	45/202 (22.3%)	0.98 (0.76 to 1.26)	0.88
A/H1N1 Michigan (2018/2019)	10/29 (34.5%)	5/27 (18.5%)	15/74 (20.3%)	17/80 (21.2%)	58/292 (19.9%)	0.93 (0.79 to 1.09)	0.37
A/H2N3 Singapore	4/23 (17.4%)	9/44 (20.5%)	17/59 (28.8%)	13/81 (16.0%)	62/295 (21.0%)	1.02 (0.87 to 1.19)	0.83
B/Maryland	1/6 (16.7%)	1/7 (14.3%)	5/31 (16.1%)	7/48 (14.6%)	91/410 (22.2%)	1.20 (0.88 to 1.62)	0.25
B/Brisbane	0/5 (0.0%)	0/2 (0.0%)	2/9 (22.2%)	4/22 (18.2%)	59/252 (23.4%)	1.38 (0.87 to 2.20)	0.18
B/Phuket	2/29 (6.9%)	7/37 (18.9%)	17/68 (25.0%)	27/147 (18.4%)	117/511 (22.9%)	1.09 (0.94 to 1.27)	0.26
Any Antigen	0/0 (0.0%)	0/2 (0.0%)	4/18 (22.2%)	7/31 (22.6%)	159/741 (21.5%)	1.06 (0.74 to 1.54)	0.74

The associations of the primary composite outcome of first cardiopulmonary hospitalization or all-cause death with seroprotection levels at 4-weeks were examined using Cox proportional hazard models. Seroprotection levels were modeled as an ordinal variable including the titer categories <40, 40-79, 80-159, 160-319, and ≥320. Analyses were based on each participant season, with the number of all participant-seasons combined. Models were adjusted for country.

© 2024 Peikert A et al. *JAMA Cardiology.*

eTable 6. Cardiopulmonary Hospitalization or All-Cause Death According to Mean Log-Titer Levels at 4 Weeks as a Continuous Measure

Antigen Type	No. of Events / Total No. (%)	Hazard Ratio (95% CI)	*P*-value
A/H2N3 Hong Kong	65/290 (22.4%)	1.12 (0.92 to 1.35)	0.27
A/H1N1 Michigan (2017/2018)	65/290 (22.4%)	0.99 (0.82 to 1.20)	0.92
A/H1N1 Michigan (2018/2019)	105/502 (20.9%)	0.89 (0.77 to 1.02)	0.10
A/H2N3 Singapore	105/502 (20.9%)	1.02 (0.89 to 1.17)	0.76
B/Maryland	105/502 (20.9%)	1.10 (0.93 to 1.31)	0.28
B/Brisbane	65/290 (22.4%)	1.20 (0.97 to 1.50)	0.10
B/Phuket	170/792 (21.5%)	1.10 (0.97 to 1.25)	0.13

The associations of the primary composite outcome of first cardiopulmonary hospitalization or all-cause death with mean log-titer levels at 4-weeks were examined using Cox proportional hazard models. Analyses were based on each participant season, with the number of all participant-seasons combined. Models were adjusted for country.

© 2024 Peikert A et al. *JAMA Cardiology.*

eFigure. Multivariate Predictors of Seroconversion Status

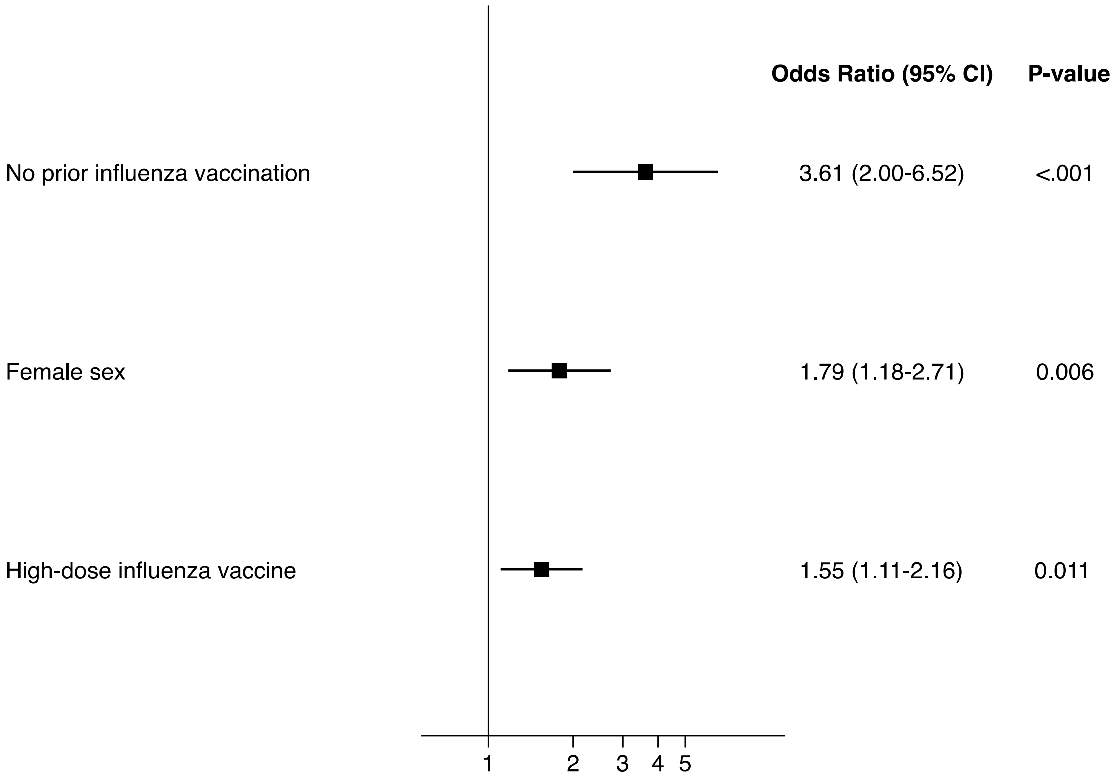

Multivariate predictors of seroconversion (defined as a ≥4-fold titer increase between baseline and 4-weeks for any of the antigens A/H2N3 Hong Kong, A/H1N1 Michigan, A/H2N3 Singapore, B/Maryland, B/Brisbane, or B/Phuket) according to patients characteristics at baseline. Estimated odds ratios and 95% confidence were derived from multivariate logistic regression. Analyses were based on the total number of participant-seasons.

© 2024 Peikert A et al. *JAMA Cardiology.*

eMethods. Vaccine Formulations by Randomized Treatment According to Influenza Season.

2017-2018 Season

High Dose Trivalent Vaccine

- A/Michigan/45/2015 X-275 (H1N1)
- A/Hong Kong/4801/2014 X-263B (H3N2)
- B/Brisbane/60/2008 (B Victoria lineage)

Standard Dose Quadrivalent Vaccine

- A/Michigan/45/2015 X-275 (H1N1)
- A/Hong Kong/4801/2014 X-263B (H3N2)
- B/Phuket/3073/2013 (B Yamagata lineage)
- B/Brisbane/60/2008 (B Victoria lineage)

2018-2019 Season

High Dose Trivalent Vaccine

- A/Michigan/45/2015 X-275 (H1N1)
- A/Singapore/INFIMH-16-0019/2016 IVR-186 (H3N2)
- B/Maryland/15/2016 BX-69A (B/Colorado/6/2017-like virus, B Victoria lineage)

Standard Dose Quadrivalent Vaccine

- A/Michigan/45/2015 X-275 (H1N1)
- A/Singapore/INFIMH-16-0019/2016 IVR-186 (H3N2)
- B/Phuket/3073/2013 (B Yamagata lineage)
- B/Maryland/15/2016 BX-69A (B/Colorado/6/2017-like virus, B Victoria lineage)

© 2024 Peikert A et al. *JAMA Cardiology.*
