# Supplementary material for: Influenza Vaccine Immune Response in Patients With High-Risk Cardiovascular Disease: A Secondary Analysis of the INVESTED Randomized Clinical Trial
Source: JAMA Cardiol. 2024 Apr 7;9(6):574–81. doi: 10.1001/jamacardio.2024.0468 (PMC11000133; doi:10.1001/jamacardio.2024.0468)
Supplement: Supplement 3. — Data sharing statement [file jamacardiol-e240468-s003.pdf]

Data Sharing Statement

Peikert. Influenza Vaccine Immune Response in Patients With High-Risk Cardiovascular Disease. *JAMA Cardiol*. Published April 07, 2024. doi:10.1001/jamacardio.2024.0468

Data
Data available: Yes
Data types: Deidentified participant data
How to access data: Data will be available on NHLBI Biolincc website
When available: With publication

Supporting Documents
Document types: None

Additional Information
Who can access the data: Data will be made available to qualified researchers as per NHLBI Guidelines
Types of analyses: Any purpose
Mechanisms of data availability: Through NHLBI Biolincc website
Any additional restrictions: None
